# Antiplasmodial Compounds from *Eurycoma harmandiana* Pierre and *Eurycoma longifolia* Jack Against Drug-Resistant *Plasmodium falciparum*: An Integrated In Vitro and In Silico Study

**DOI:** 10.3390/ijms27177892

**Published:** 2026-09-04

**Authors:** Atthaphon Konyanee, Habibah A. Wahab, Ezatul Ezleen Kamarulzaman, Ahmad Marwazi Mohd Suhaimi, Ahmad Ghazali Ismail, Prapaporn Chaniad, Walaiporn Plirat, Arisara Phuwajaroanpong, Thaweesak Juengwatanatrakul, Tripetch Kanchanapoom, Gorawit Yusakul, Chuchard Punsawad

**Affiliations:** 1 Department of Medical Sciences, School of Medicine, Walailak University, Nakhon Si Thammarat 80160, Thailand; atthaphon.ko@wu.ac.th (A.K.); prapaporn.ch@wu.ac.th (P.C.); walaiporn.pi@wu.ac.th (W.P.); 2 Center of Excellence in Tropical Pathobiology, Walailak University, Nakhon Si Thammarat 80160, Thailand; arisara.pw@wu.ac.th; 3 School of Pharmaceutical Sciences, Universiti Sains Malaysia, Minden 11800, Pulau Pinang, Malaysia; habibahw@usm.my (H.A.W.); ezatulezleen@usm.my (E.E.K.); ahmadmarwazi98@gmail.com (A.M.M.S.); ghazali.upha17@student.usm.my (A.G.I.); 4 Malaysian Institute of Pharmaceuticals and Nutraceuticals, National Institutes of Biotechnology Malaysia, Halaman Bukit Gambir, Gelugor 11700, Pulau Pinang, Malaysia; 5 Faculty of Pharmacy, Universitas Airlangga, Kampus C UNAIR, Jl. Mulyorejo, Mulyorejo, Surabaya 60115, East Java, Indonesia; 6 School of Allied Health Sciences, Walailak University, Nakhon Si Thammarat 80160, Thailand; 7 Faculty of Pharmaceutical Sciences, Ubon Ratchathani University, Ubon Ratchathani 34190, Thailand; thaweesak.j@ubu.ac.th; 8 Faculty of Pharmaceutical Sciences, Khon Kaen University, Khon Kaen 40002, Thailand; trikan@kku.ac.th; 9 Faculty of Pharmaceutical Sciences, Naresuan University, Phitsanulok 65000, Thailand; gorawity@nu.ac.th; 10 Research and Innovation Cluster for Natural Health Products, Naresuan University, Phitsanulok 65000, Thailand

**Keywords:** antiplasmodial, *Eurycoma harmandiana*, *Eurycoma longifolia*, molecular docking, molecular dynamics simulations

## Abstract

Malaria is a life-threatening global disease, and despite artemisinin-based combination therapies (ACTs) as first-line treatment, emerging drug-resistant *Plasmodium* strains necessitate novel antimalarial agents. This study investigated the antiplasmodial potential of *Eurycoma harmandiana* Pierre (EH) root extract, a medicinal plant closely related to *Eurycoma longifolia* Jack (EL). The extract and its derived compounds were evaluated using in vitro antiplasmodial and cytotoxicity assays. The active compounds were further investigated by parasite morphological analysis, molecular docking against quadruple-mutant *Plasmodium falciparum* dihydrofolate reductase (qm*Pf*DHFR), molecular dynamics (MD) simulations, and in silico prediction of drug-likeness, pharmacokinetic properties, and toxicity. The ethanolic extract exhibited potent antiplasmodial activity (IC_50_ = 0.51 µg/mL) with low cytotoxicity (CC_50_ = 31.68 µg/mL) and a high selectivity index (SI = 62.11). Quassinoids showed the strongest activity (IC_50_ = 0.13–0.87 µM), whereas alkaloids displayed good to moderate activity. The extract and two promising bioactive quassinoids, eurycomanone (**1**) and glaucarubolone (**5**), disrupted intraerythrocytic parasite development. Molecular docking and MD simulations demonstrated that glaucarubolone (**5**) exhibited favorable predicted interactions with qm*Pf*DHFR, along with favorable predicted drug-like properties, pharmacokinetic profiles, and low toxicity. This study provides the first report of the antiplasmodial activity of *Eurycoma harmandiana*, highlighting it as a promising alternative source of bioactive compounds against *Plasmodium* parasites. Glaucarubolone (**5**) may represent a promising scaffold for further investigation toward the development of novel antimalarial agents.

## 1. Introduction

*Plasmodium falciparum* is the most virulent species responsible for malaria in humans. In 2024, the number of malaria cases was estimated at 282 million globally, with approximately 610,000 deaths [1]. The World Health Organization (WHO) recommends artemisinin-based combination therapies (ACTs) as the first-line treatment for uncomplicated *P. falciparum* [2]. However, partial artemisinin resistance has emerged in several malaria-endemic regions and was first reported in the Greater Mekong Subregion (GMS), followed by subsequent reports from other parts of the world [3]. This partial resistance is associated with non-synonymous mutations in the propeller domain of the *Plasmodium falciparum kelch13* (*Pfkelch13*) gene and is clinically defined by delayed parasite clearance and increased rates of treatment failure [3,4]. In Thailand, dihydroartemisinin–piperaquine was withdrawn from the Sisaket and Ubon Ratchathani provinces because of the high treatment failure rates observed through the Integrated Drug Efficacy Surveillance, and artesunate–pyronaridine was subsequently introduced as the first-line treatment in these provinces [5]. The emergence and spread of antimalarial drug resistance highlight the urgent need for the development of novel antimalarial therapies. Plants represent valuable sources of bioactive compounds with potential for antimalarial drug development [6]. The alkaloid quinine, derived from the bark of the *Cinchona* species, later inspired the development of chloroquine [7]. Similarly, naphthoquinone lapachol, derived from the *Tabebuia* species, serves as the lead compound for the synthesis of atovaquone [8]. Furthermore, the sesquiterpene lactone endoperoxide artemisinin, derived from the Chinese medicinal plant *Artemisia annua* L., provides a key scaffold for the development of improved artemisinin derivatives with enhanced antimalarial efficacy and improved oral bioavailability [6].

*Eurycoma longifolia* Jack (EL), a member of the Simaroubaceae family, is a tall, slender shrub native to Southeast Asia, particularly Malaysia, Indonesia, and Vietnam. It is also found in certain regions of Cambodia, Myanmar, and Thailand [9]. This plant is locally known as “Tongkat Ali” in Malaysia and “Pla Lai Phueak Yai” in Thailand. EL roots have been traditionally used for various medicinal purposes, including antimalarial, antipyretic, and aphrodisiac applications [10]. EL decoction has been traditionally valued for its aphrodisiac properties for centuries in Malaysia and other parts of Southeast Asia and is now considered a national treasure plant in Malaysia [9,11]. Several studies have demonstrated that EL extracts and their constituents exhibit diverse pharmacological activities, including antiplasmodial, anticancer, antidiabetic, and antimicrobial effects [9]. In contrast, *Eurycoma harmandiana* Pierre (EH) has received comparatively limited scientific attention. This species is native to northeastern Thailand and Laos and is commonly known in Thailand as “Pla Lai Phueak Lek” [12,13]. The roots of EH are in high demand as an aphrodisiac herbal supplement and are also utilized in Thai traditional medicine for the treatment of intermittent fever (malaria) [12,14]. Several studies have reported that EH and its bioactive constituents exhibit a range of pharmacological activities, including anti-inflammatory [12,15], anti-erectile dysfunction [16], anti-cancer [17], and anti-SARS-CoV-2 [18]. Our research group has successfully isolated quassinoids, canthin-6-one alkaloids, and *β*-carboline alkaloids from the intact roots of EH [19,20]. Although EL and EH possess distinct botanical characteristics, both species produce similar classes of compounds, including quassinoids, canthin-6-one alkaloids, and *β*-carboline alkaloids [13]. However, research on EH remains limited compared to that on EL, with a notable lack of studies investigating the antiplasmodial activity of its extracts and derived compounds.

*Plasmodium falciparum* dihydrofolate reductase (*Pf*DHFR) is an essential enzyme involved in the de novo folate biosynthesis pathway that plays a critical role in the generation of purines, pyrimidines, and certain amino acids that are crucial for parasite cell growth and proliferation [21]. *Pf*DHFR catalyzes the reduction of dihydrofolate (DHF) to tetrahydrofolate (THF) using NADPH as a cofactor [21]. During DNA synthesis, folate in the form of 5,10-methylenetetrahydrofolate (5,10-methylene-THF) serves as a one-carbon donor for the methylation of deoxyuridine monophosphate (dUMP) to deoxythymidine monophosphate (dTMP) in a reaction catalyzed by thymidylate synthase (TS) [22]. The triphosphate form of dTMP (dTTP) is subsequently incorporated into the growing DNA strand during DNA replication. Antifolate drugs targeting *Pf*DHFR, such as cycloguanil and pyrimethamine, have long been used in combination with sulfonamide drugs for the treatment and prophylaxis of malaria [23]. Notably, quadruple mutations N51I, C59R, S108N, and I164L induce conformational changes in the active site of *Pf*DHFR, thereby reducing drug binding affinity [24]. These mutations are associated with clinical failure of sulfadoxine–pyrimethamine (SP) therapy [25]. Although several qm*Pf*DHFR inhibitors, including WR99210, P65, and P218, have been developed [26], the identification of novel compounds with activity against resistant *Pf*DHFR variants remains an important strategy for antimalarial drug development.

Given the close relationship between EL and EH and their shared phytochemical constituents, EH may represent a potential alternative source of antiplasmodial compounds. Therefore, this study evaluated the in vitro antiplasmodial activity and cytotoxicity of root extracts and derived compounds from EL and EH, together with their interactions with qm*Pf*DHFR through molecular docking and MD simulations. To the best of our knowledge, this is the first study to investigate the antiplasmodial activity of EH. These findings may support the further development and standardization of EH extracts and provide preliminary insights for future investigations into their antimalarial mechanisms of action, as well as the rational design of potential antimalarial scaffolds.

## 2. Results

### 2.1. In Vitro Antiplasmodial Activity of Plant Extracts and Compounds Derived from EL and EH Roots

The antiplasmodial activity of plant extracts and compounds derived from EL and EH roots against the drug-resistant *P. falciparum* K1 strain is shown in Table 1. The antiplasmodial activity classification of plant extracts was defined in a previous study as follows: high activity (IC_50_ ≤ 5 μg/mL), promising activity (5 μg/mL < IC_50_ ≤ 15 μg/mL), moderate activity (15 μg/mL < IC_50_ ≤ 50 μg/mL), and weak activity (50 μg/mL < IC_50_ < 100 μg/mL) [27]. Notably, the ethanolic extract of EH roots (EEHR) displayed the lowest IC_50_ value among the plant extracts at less than 1 µg/mL (0.51 ± 0.10 μg/mL), followed by the ethanolic extract of EL roots (EELR) and aqueous extract of EL roots (AELR) with IC_50_ values of 1.21 ± 1.03 and 1.40 ± 0.46 μg/mL, respectively. Among the observed extracts, the aqueous extract of EH roots (AEHR) exhibited the highest IC_50_ value at 3.09 ± 0.77 μg/mL (Table 1). Therefore, all the extracts exhibited high antiplasmodial activity. Notably, the antiplasmodial activity of EEHR was approximately 2.37-fold higher than that of EELR. AELR exhibited approximately 2.20-fold more potency than AEHR when comparisons were made within the same extraction solvent system.

Compounds derived from EL and EH roots were classified based on previous studies as follows: excellent activity (IC_50_ < 1 μM), good activity (IC_50_ 1–20 μM), moderate activity (IC_50_ 20–100 μM), weak activity (IC_50_ 100–200 μM), and inactive (IC_50_ > 200 μM) [28]. Regarding the C-20 quassinoid compounds, the lowest IC_50_ value was observed for glaucarubolone (**5**) (0.13 ± 0.03 μM), which was closely comparable to that of eurycomanone (**1**) (0.14 ± 0.07 μM) and 13α(21)-epoxyeurycomanone (**2**) (0.16 ± 0.08 μM), followed by that of chaparrinone (**4**) (0.27 ± 0.03 μM). In contrast, the C-19 quassinoid, eurycomalactone (**3**), displayed the highest IC_50_ value (0.87 ± 0.16 μM). Overall, all bioactive quassinoid compounds exhibited excellent in vitro antiplasmodial activity.

For the alkaloid compounds, 9-methoxycanthin-6-one (**7**) exhibited a higher IC_50_ value than the quassinoids at 16.54 ± 1.16 μM and was classified as having good antiplasmodial activity, being approximately 127.23-fold less potent than glaucarubolone (**5**). This was followed by canthin-6-one 9-O-*β*-glucopyranoside (**8**), *β*-carboline-1-propionic acid (**9**), and canthin-6-one (**6**), with IC_50_ values of 20.15 ± 4.75, 35.32 ± 9.20, and 47.31 ± 10.88 μM, respectively. These compounds exhibited moderate antiplasmodial activity. Compared with the reference antimalarial drugs, artesunate and chloroquine, all quassinoids exhibited lower IC_50_ values than chloroquine (IC_50_ = 0.89 ± 0.26 μM), corresponding to an approximately 1.02 to 6.84-fold greater potency relative to chloroquine. However, all quassinoids were 13 to 87-fold less potent than artesunate (0.01 ± 0.00 μM). In contrast, all alkaloid compounds were less potent than chloroquine and artesunate.

### 2.2. In Vitro Cytotoxicity and SI of Plant Extracts and Compounds Derived from EL and EH Roots

The cytotoxicity of plant extracts and compounds derived from EL and EH roots was evaluated against mammalian Vero cells (African green monkey kidney epithelial cells), as summarized in Table 1. The SI was calculated to determine the selectivity toward malarial parasites over mammalian cells. SI was defined as the ratio of the 50% cytotoxic concentration (CC_50_) against Vero cells to the IC_50_ value against the *P. falciparum* K1 strain. Higher SI values indicate greater selectivity toward the parasite. Based on previously reported criteria, SI values greater than 10 were considered indicative of a favorable safety window [29].

Among the plant extracts, EELR exhibited the highest CC_50_ value (CC_50_ > 100 µg/mL), followed by AEHR, AELR, and EEHR, with CC_50_ values of 72.41 ± 9.25, 53.81 ± 9.36, and 31.68 ± 5.75 µg/mL, respectively. In addition, EELR demonstrated the highest SI against *P. falciparum* K1 strain (>82.64), followed by EEHR (62.11). The SI values for AELR and AEHR were 38.43 and 23.43, respectively. All plant extracts demonstrated greater selectivity toward the parasite than toward Vero cells, with SI values exceeding 10.

For quassinoid compounds, eurycomanone (**1**) demonstrated the highest CC_50_ value (28.84 ± 6.18 µM) and the highest SI value (206.00), indicating strong selectivity toward the malaria parasite. This was followed by 13α(21)-epoxyeurycomanone (**2**) (CC_50_ = 11.28 ± 1.73 µM, SI = 70.50), glaucarubolone (**5**) (CC_50_ = 6.11 ± 2.20 µM, SI = 47.00), and chaparrinone (**4**) (CC_50_ = 6.71 ± 3.83 µM, SI = 24.85). In contrast, eurycomalactone (**3**) exhibited the lowest CC_50_ value among the quassinoids (5.97 ± 1.54 µM) and the lowest SI value (6.86). Thus, eurycomanone (**1**), 13α(21)-epoxyeurycomanone (**2**), chaparrinone (**4**), and glaucarubolone (**5**) demonstrated SI values greater than 10, whereas eurycomalactone (**3**) demonstrated limited selectivity. Doxorubicin (positive control) exhibited the lowest CC_50_ value among all the tested compounds (4.28 ± 2.81 µM). Among alkaloid compounds, *β*-carboline-1-propionic acid (**9**) demonstrated the highest CC_50_ value, exceeding the highest tested concentration (CC_50_ > 416 µM), with an SI value greater than 11.77. This was followed by canthin-6-one 9-O-*β*-glucopyranoside (**8**), canthin-6-one (**6**), and 9-methoxycanthin-6-one (**7**), with CC_50_ values of 39.07 ± 9.76, 36.01 ± 6.11, and 21.95 ± 3.09 µM, respectively. The SI values of canthin-6-one 9-O-*β*-glucopyranoside (**8**), 9-methoxycanthin-6-one (**7**), and canthin-6-one (**6**) were 1.93, 1.32, and 0.76, respectively, indicating low selectivity toward the parasite.

Overall, the SI values were used to guide the selection of plant extracts and compounds for further evaluation as potential antimalarial agents. Based on their in vitro antiplasmodial activity (low IC_50_ values) and favorable selectivity (SI values greater than 10), AELR, EELR, AEHR, and EEHR were selected for further evaluation. Among the isolated compounds, eurycomanone (**1**) and glaucarubolone (**5**), representing the two most promising C-20 quassinoids based on their antiplasmodial activity and selectivity, were chosen for comparison with the corresponding extracts. These selected extracts and compounds were subsequently evaluated for their effects on parasite morphology during intraerythrocytic development.

### 2.3. Morphological Changes in P. falciparum K1 Strain-Infected Erythrocytes Treated with Plant Extracts and Promising C-20 Quassinoid Compounds

**Figure 1:**
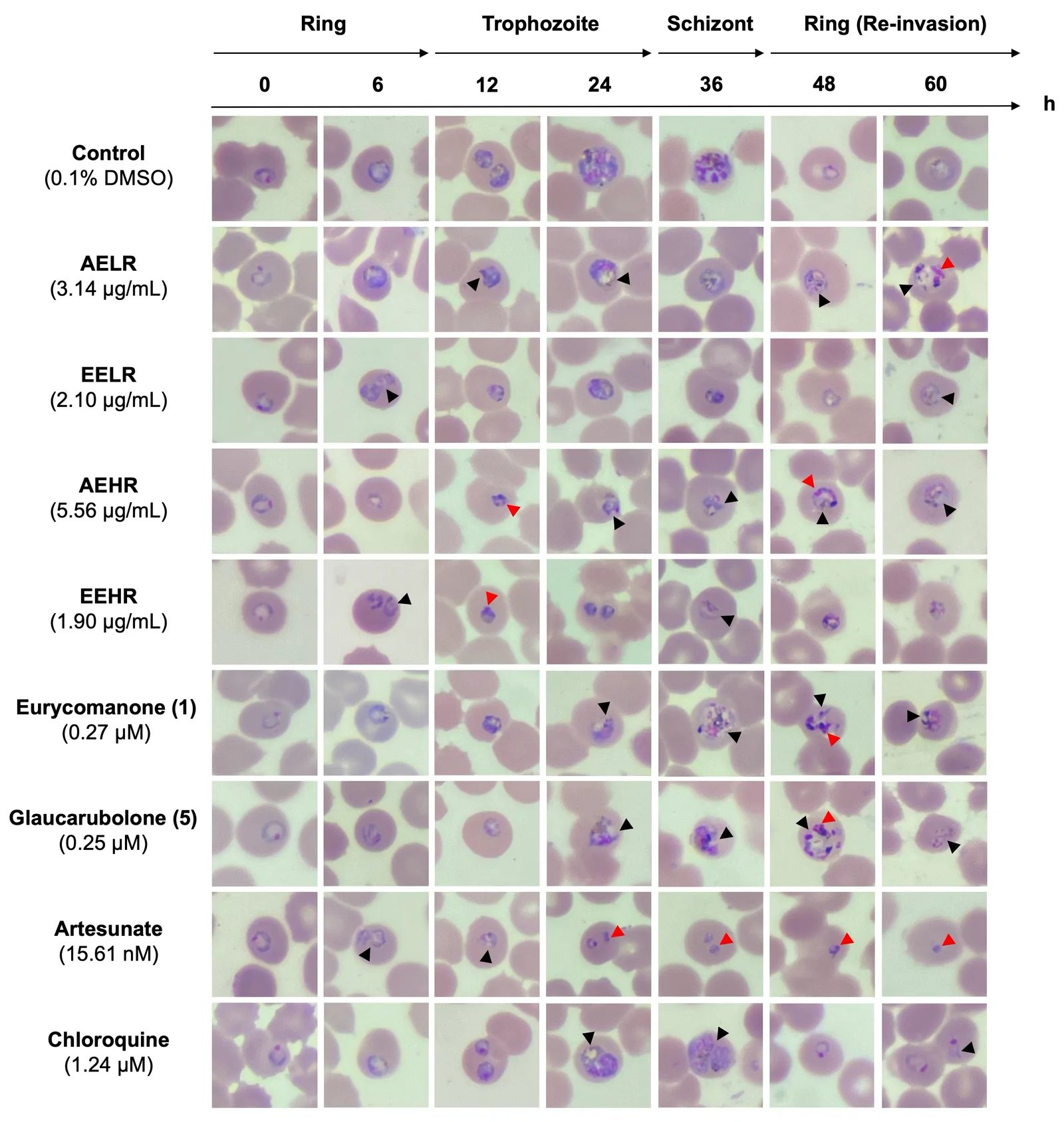
Giemsa-stained thin blood smears of the *P. falciparum* K1 strain after exposure to IC_90_ concentrations of plant extracts/compounds compared with the control at 0 (baseline), 6, 12, 24, 36, 48, and 60 h post-treatment. Treatments included plant extracts (AELR, EELR, AEHR, and EEHR), the two most promising compounds (eurycomanone (**1**) and glaucarubolone (**5**)), and reference antimalarial drugs (artesunate and chloroquine). Each panel demonstrates the predominant parasite stage observed after treatment or in the control. Morphological changes are indicated by red arrowheads for nuclear abnormalities and black arrowheads for cytoplasmic abnormalities. The Giemsa-stained thin blood smears were examined using a 100× oil immersion objective lens.

The effects of extracts and compounds derived from EL and EH roots on *P. falciparum* K1 strain morphology are shown in Figure 1. EELR and EEHR induced noticeable early-stage morphological alterations after 6 h of exposure, particularly in ring-stage parasites, characterized by cytoplasmic abnormalities (black arrowheads). In addition, EEHR induced nuclear abnormalities at 12 h (red arrowheads). Following the initial morphological changes observed at 6 h, both EELR- and EEHR-treated parasites exhibited progressive shrinkage, and their development was arrested throughout the 60 h observation period compared with the control. In cultures treated with AELR and AEHR, pronounced morphological changes were first observed at 12 h during the early trophozoite stage, with the presence of both cytoplasmic (black arrowheads) and nuclear (red arrowheads) changes, which persisted throughout the 60-h incubation period compared with the control. In the compound-treated groups, eurycomanone (**1**) and glaucarubolone (**5**) suppressed the parasite growth at 24 h during the trophozoite stage, with cytoplasmic (black arrowheads) and nuclear (red arrowheads) abnormalities. At 48 h, the treated parasites failed to fully develop to the ring stage (re-invasion) compared with the control. For artesunate, morphological effects were evident at 6 h in early ring-stage parasites, demonstrating cytoplasmic abnormalities (black arrowheads) and persistent nuclear abnormalities (pyknotic) observed from 24 to 60 h. In contrast, chloroquine treatment resulted in cytoplasmic abnormalities in the trophozoite stage at 24 and 36 h (black arrowheads). However, most parasites developed into the ring stage at 48 h and remained in the ring stage at 60 h, unlike the control, which progressed to early trophozoites. Interestingly, parasites in the chloroquine-treated group were comparable in size to those in the control group, particularly when compared with parasites treated with plants, quassinoid compounds, or artesunate, which demonstrated pronounced growth inhibition.

Overall, EELR and EEHR affected parasite growth at the early-ring stage as early as 6 h, whereas AELR, AEHR, eurycomanone (**1**), and glaucarubolone (**5**) primarily affected parasite growth at the trophozoite stage between 12 and 24 h. However, all extracts and C-20 quassinoid compounds markedly inhibited the development of the drug-resistant *P. falciparum* K1 parasite to the mature schizont stage. In addition, all extracts and compounds markedly reduced parasitemia at 48 h (re-invasion stage) because the parasites failed to develop into schizonts containing daughter merozoites. Consequently, parasitemia was substantially lower than that in the control group, reaching an average of 3.11% (Supplementary Figure S1). At 48 h, cultures treated with extracts or compounds demonstrated parasitemia, ranging from an average of 0.81–1.05%, which was slightly less potent than artesunate (average 0.64%) but markedly lower than chloroquine (average 2.01%) (Supplementary Figure S1).

### 2.4. Molecular Docking Analysis of Compounds Derived from EL and EH Root Interaction with qmPfDHFR

The binding energies and protein–ligand interactions involving key amino acid residues of compounds derived from EL and EH roots against qm*Pf*DHFR are summarized in Table 2. Redocking of the known qm*Pf*DHFR inhibitor WR99210 yielded an RMSD of 0.608 Å, which is below the commonly accepted threshold of 2.00 Å. This result indicates good agreement between the redocked and crystallographic binding conformations, thereby supporting the reliability of the molecular docking protocol (Figure S2).

**Figure 2:**
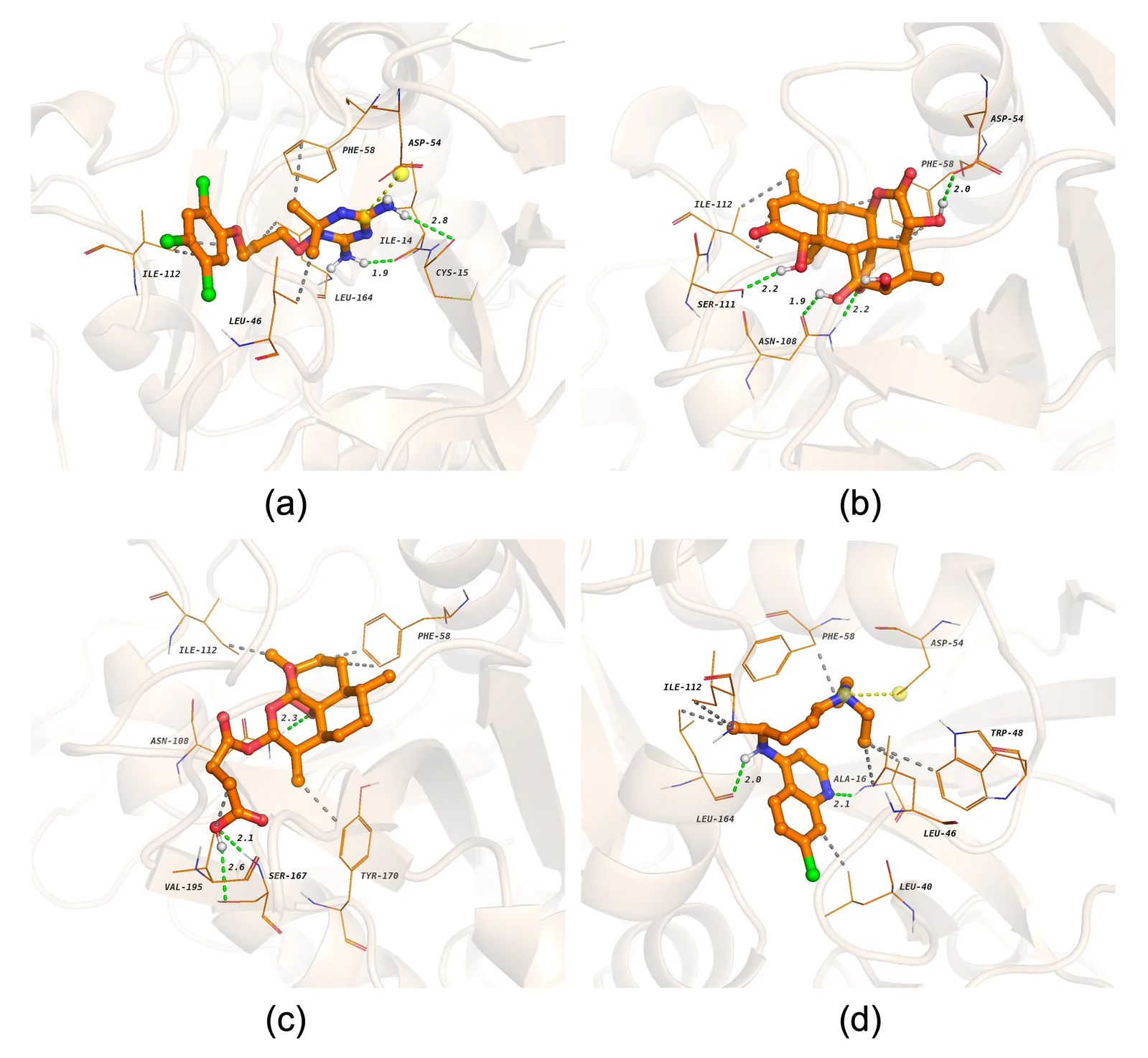
Intermolecular interactions of promising compounds and antimalarial drugs with qm*Pf*DHFR. Ligands include WR99210 (co-crystallized ligand/qm*Pf*DHFR inhibitor) (**a**), glaucarubolone (**5**) (**b**), artesunate (**c**), and chloroquine (**d**). Ligands are depicted as ball-and-stick models, while interacting residues are shown as line models. Heteroatoms are color-coded as follows: carbon (C), orange; oxygen (O), red; nitrogen (N), blue; chlorine (Cl), green; sulfur (S), yellow; and hydrogen (H), white. Green dashed lines indicate hydrogen bonds, with bond distances labeled in angstroms (Å). Gray dashed lines represent hydrophobic interactions, whereas yellow dashed lines and spheres denote salt-bridge interactions.

**Figure 3:**
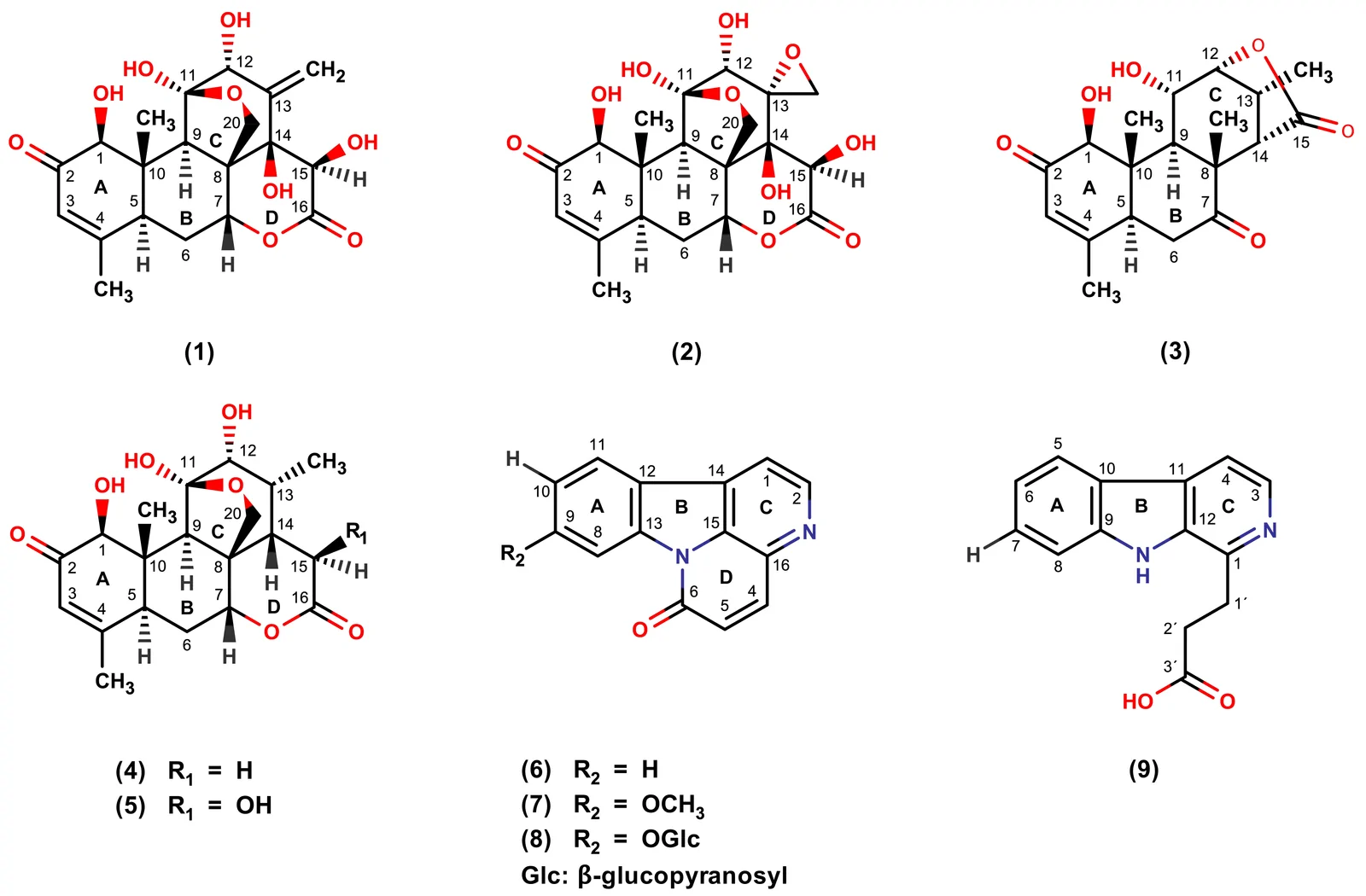
Chemical structures of quassinoids (**1**)–(**5**), canthin-6-one alkaloids (**6**)–(**8**), and *β*-carboline alkaloid (**9**) derived from EL and EH roots.

Among the tested compounds, the C-20 quassinoid glaucarubolone (**5**) exhibited the highest binding affinity (−9.07 kcal/mol). Glaucarubolone (**5**) formed hydrogen bonds with ASP54, ASN108 (two interactions), and SER111 and hydrophobic interactions with PHE58 (three interactions) and ILE112 (two interactions) (Figure 2b). Chaparrinone (**4**) demonstrated the second-highest binding affinity (−8.77 kcal/mol), forming hydrogen bonds with ASN108 (two interactions) and SER111 and hydrophobic interactions with PHE58 (three interactions) and ILE112 (Supplementary Figure S3d). The higher binding affinity toward qm*Pf*DHFR of glaucarubolone (**5**) compared with chaparrinone (**4**) is likely attributable to the presence of a hydroxyl group at the C-15 position, suggesting a preliminary structure–activity relationship (SAR) associated with C-15 hydroxyl substitution (Figure 3).

Other C-20 quassinoids, including eurycomanone (**1**) (−8.27 kcal/mol) and 13α(21)-epoxyeurycomanone (**2**) (−8.04 kcal/mol), demonstrated slightly lower binding affinities toward qm*Pf*DHFR. The presence of an additional hydroxyl group at the C-14 position (Figure 3) did not appear to significantly enhance the binding interactions with qm*Pf*DHFR. Furthermore, the C-19 quassinoid eurycomalactone (**3**) demonstrated favorable binding affinity (−8.19 kcal/mol) and uniquely formed a hydrogen bond with LEU46, a residue not observed in the binding patterns of the C-20 quassinoids (Supplementary Figure S3c).

Among the alkaloid class, canthin-6-one 9-O-*β*-glycopyranoside (**8**) exhibited the highest binding affinity (−8.28 kcal/mol). It formed hydrogen bonds with ILE14 (two interactions), ASP54, ASN108, LEU164, and TYR170, as well as hydrophobic interactions involving VAL45, PRO113, and PHE116 (Supplementary Figure S3g). In contrast, 9-methoxycanthin-6-one (**7**) (−6.70 kcal/mol) and canthin-6-one (**6**) (−6.43 kcal/mol) displayed weaker binding affinities. The enhanced binding affinity of canthin-6-one 9-O-*β*-glycopyranoside (**8**) may suggest that *β*-glycopyranosyl substitution at the C-9 position improves its interaction with qm*Pf*DHFR, indicating the importance of C-9 functionalization within the canthin-6-one scaffold (Figure 3). *β*-carboline-1-propionic acid (**9**) demonstrated moderate binding affinity (−7.19 kcal/mol), forming hydrogen bonds with ALA16, ASN108 (two interactions), and LEU164 and hydrophobic interactions with LEU40 (two interactions), LEU46, PHE58, and ASN108 (Supplementary Figure S3h).

The WR99210, a known qm*Pf*DHFR inhibitor, demonstrated a binding energy of −7.64 kcal/mol, forming hydrogen bonds with ILE14 and CYS15; hydrophobic interactions with LEU46, PHE58, ILE112 (two interactions), and LEU164; and a salt bridge interaction with ASP54 (Figure 2a). Common interacting residues shared among the derived compounds included ASP54, PHE58, ASN108, SER111, and ILE112 for quassinoids and LEU46, ASN108, ILE112, and PHE116 for canthin-6-one and *β*-carboline alkaloids. These residues are located within the qm*Pf*DHFR active site and are involved in WR99210 binding, indicating a conserved binding region targeted by both the reference inhibitor and plant-derived compounds.

Molecular docking analysis of reference antimalarial drugs revealed that artesunate exhibited favorable binding affinity (−8.50 kcal/mol), forming hydrogen bonds with ASN108 and SER167 (two interactions), and hydrophobic interactions with PHE58 (two interactions), ILE112, TYR170, and VAL195 (Figure 2c). In contrast, chloroquine displayed weaker binding affinity (−7.49 kcal/mol), forming hydrogen bonds with ALA16 and LEU164, multiple hydrophobic interactions, and a salt bridge interaction with ASP54, similar to that observed for WR99210 (Figure 2d).

Overall, several compounds derived from EL and EH roots demonstrated favorable binding interactions with qm*Pf*DHFR, particularly with the quassinoid compounds displaying particularly strong binding affinities, all of which exhibited binding energies below −8.0 kcal/mol. Based on both molecular docking results and in vitro antiplasmodial activity (IC_50_ = 0.13 ± 0.03 µM), glaucarubolone (**5**) was selected for further investigation as a representative quassinoid compound. Its superior binding affinity toward qm*Pf*DHFR (−9.07 kcal/mol), which exceeded that of the reference qm*Pf*DHFR inhibitor WR99210, supports its selection for MD simulations to assess ligand–protein complex stability under dynamic conditions.

### 2.5. MD Simulations Analysis of Promising Compounds Derived from EL and EH Roots in Complex with qmPfDHFR

**Figure 4:**
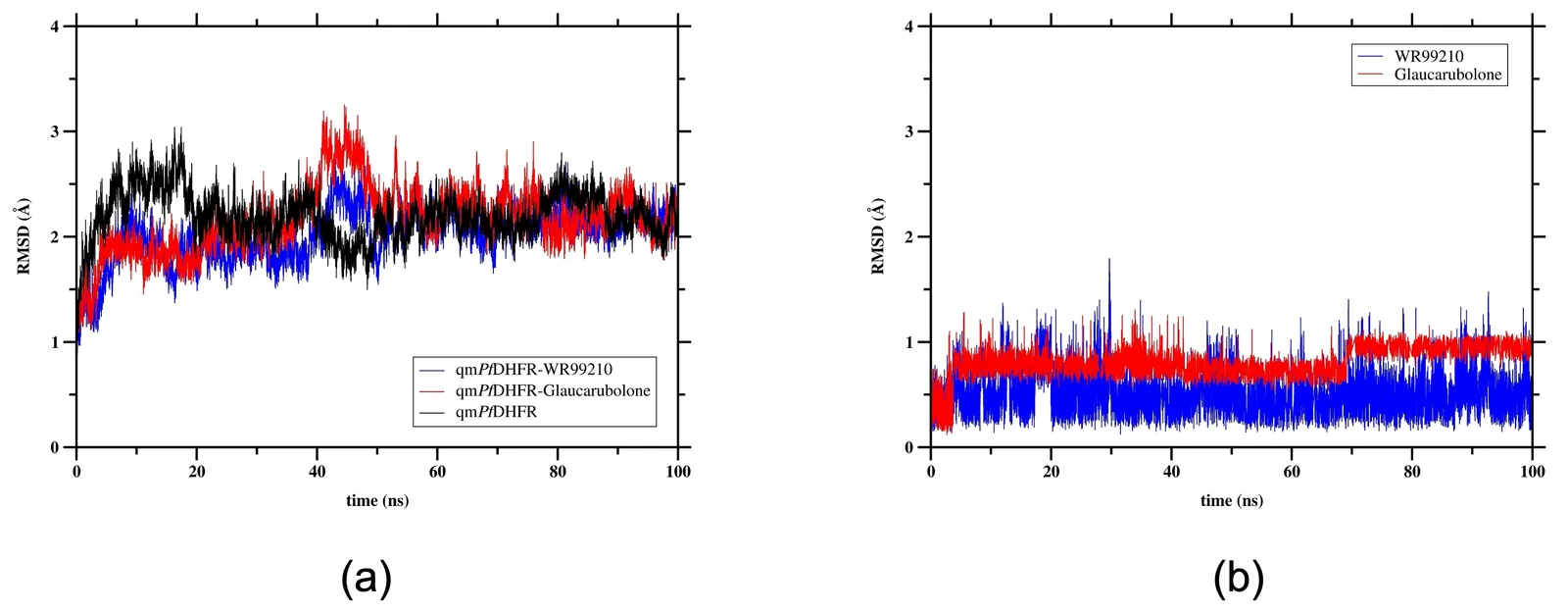
Root mean square deviation (RMSD) profiles of apo qm*Pf*DHFR and ligands complexed with qm*Pf*DHFR. (**a**) RMSD of the enzyme backbone in complex with the qm*Pf*DHFR inhibitor WR99210 (blue), the promising compound glaucarubolone (**5**) (red), and apo qm*Pf*DHFR (black) throughout the 100 ns MD simulations. (**b**) RMSD of ligands WR99210 (blue) and glaucarubolone (**5**) (red) within the qm*Pf*DHFR binding site over the same 100 ns simulations.

**Figure 5:**
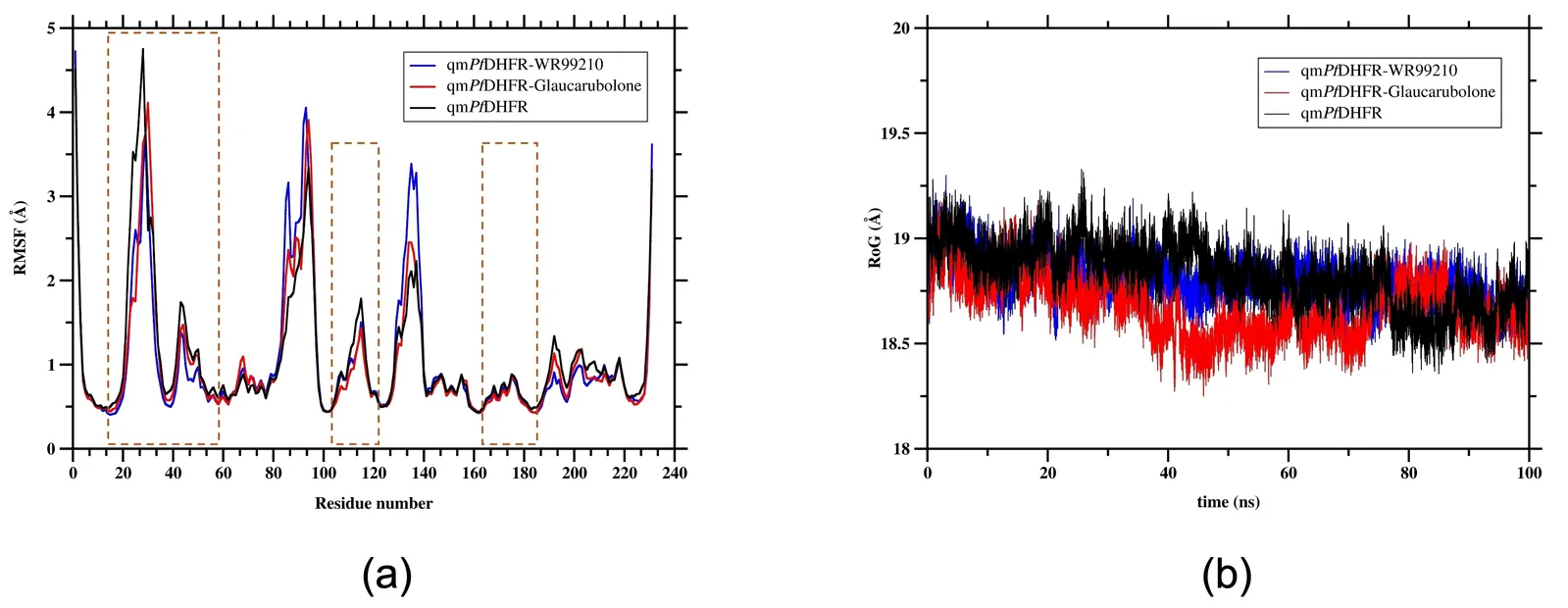
Root mean square fluctuation (RMSF) and radius of gyration (RoG) profiles of apo qm*Pf*DHFR and qm*Pf*DHFR complexes throughout the 100 ns MD simulation. (**a**) RMSF values indicate residue-level fluctuations of qm*Pf*DHFR backbone atoms in complex with the qm*Pf*DHFR inhibitor WR99210 (blue), the promising compound glaucarubolone (**5**) (red), and apo qm*Pf*DHFR (black). The orange dashed box indicates the ligand-binding site residues. (**b**) The RoG of qm*Pf*DHFR backbone atoms in complex with WR99210 (blue), glaucarubolone (**5**) (red), and apo qm*Pf*DHFR (black) over the 100 ns simulation period.

The structural stability and conformational dynamics of qm*Pf*DHFR in both the apo and ligand-bound forms were evaluated using RMSD, RMSF, and RoG analyses throughout the 100 ns MD simulations, as shown in Figure 4 and Figure 5. The corresponding mean RMSD, RMSF, and RoG values are summarized in Table 3. The simulations were performed using the cofactor-free form of the qm*Pf*DHFR structure in accordance with previously reported studies [30,31].

The RMSD analysis was performed to assess the conformational stability of the ligand-bound qm*Pf*DHFR complexes compared with apo qm*Pf*DHFR by monitoring Cα backbone deviations throughout the 100 ns simulations [30]. As shown in Figure 4a, the RMSD of the qm*Pf*DHFR–WR99210 complex increased from approximately 1.0 Å to 2.0 Å within the first 10 ns, remained relatively stable until 40 ns, and subsequently increased to approximately 2.5 Å between 40 and 50 ns before decreasing and remaining relatively stable for the remainder of the simulation. The overall mean RMSD was 1.64 ± 0.34 Å (Table 3). A comparable trend was observed for the qm*Pf*DHFR–glaucarubolone (**5**) complex, which exhibited a transient increase to approximately 3.0 Å between 40 and 50 ns, followed by a decrease and subsequent stabilization for the remainder of the simulation. The overall mean RMSD was 1.70 ± 0.28 Å, slightly higher than that observed for the WR99210-bound complex (Figure 4a). In contrast, apo qm*Pf*DHFR displayed a different RMSD profile, with a higher overall mean RMSD of 2.14 ± 0.37 Å compared with the ligand-bound complexes (Table 3). The lower RMSD values observed for the glaucarubolone (**5**)–qm*Pf*DHFR complex suggest that the ligand-bound protein reduced the overall structural deviation of qm*Pf*DHFR compared with the apo qm*Pf*DHFR throughout the simulation. Ligand RMSD analysis further showed that glaucarubolone (**5**) exhibited a slightly higher mean RMSD of 0.63 ± 0.22 Å than WR99210 (0.44 ± 0.16 Å), indicating slightly greater positional fluctuations of glaucarubolone (**5**) within the qm*Pf*DHFR binding site (Figure 4b).

RMSF analysis was performed to evaluate the residue-level flexibility and conformational dynamics of qm*Pf*DHFR in complex with WR99210 and glaucarubolone (**5**) compared to the apo-qm*Pf*DHFR. Higher RMSF values indicate greater residue mobility, whereas lower values reflect reduced conformational fluctuations [30,32]. As shown in Figure 5a, qm*Pf*DHFR residues exhibited fluctuations in the range of approximately 0.4 to 4.7 Å throughout the simulations, with pronounced peaks observed in three distinct regions across all three systems. The mean RMSF values were 1.09 ± 0.77 Å for apo qm*Pf*DHFR, 1.06 ± 0.81 Å for the WR99210–qm*Pf*DHFR complex, and 1.02 ± 0.75 Å for the glaucarubolone (**5**)–qm*Pf*DHFR complex, indicating that ligand binding had minimal impact on the overall residue flexibility (Table 3). Residues involved in inhibitor binding or located within the active site, including ILE14, CYS15, ALA16, LEU46, TRP48, ASP54, MET55, TYR57, PHE58, VAL103, MET104, ASN108, SER111, ILE112, PRO113, PHE116, ARG122, LEU164, GLY165, TYR170, TYR183, and THR185, generally exhibited lower fluctuations in the ligand-bound systems than in apo qm*Pf*DHFR, particularly within the ligand-binding region indicated by the orange dashed box (Figure 5a). Notably, the glaucarubolone (**5**)–qm*Pf*DHFR complex showed reduced fluctuations, particularly at ASN108 (0.70 Å), SER111 (0.94 Å), and ILE112 (0.94 Å) compared with both the WR99210-bound and apo systems (Figure 5a). Overall, the slightly lower mean RMSF value observed for the glaucarubolone (**5**)–qm*Pf*DHFR complex, together with reduced fluctuations at several residues involved in ligand binding, suggests that glaucarubolone (**5**) may contribute to reduced local flexibility within the qm*Pf*DHFR binding site during the simulation.

The RoG analysis was performed to evaluate the overall compactness of qm*Pf*DHFR during the 100 ns MD simulations, with lower RoG values indicating a more compact protein conformation [30,33]. As shown in Figure 5b, all three systems exhibited highly comparable mean RoG values of 18.83 ± 0.15 Å, 18.80 ± 0.10 Å, and 18.68 ± 0.14 Å for apo qm*Pf*DHFR, the WR99210–qm*Pf*DHFR complex, and the glaucarubolone (**5**)–qm*Pf*DHFR complex, respectively (Table 3). Among the three systems, the glaucarubolone (**5**)–qm*Pf*DHFR complex exhibited the lowest mean RoG value, suggesting enhanced structural compactness relative to the WR99210-bound and apo systems. Overall, the comparable RoG values across all three systems indicate that the global structural integrity of qm*Pf*DHFR was largely maintained throughout the simulations, while the glaucarubolone (**5**)–qm*Pf*DHFR complex displayed a modest tendency toward a more compact conformation.

### 2.6. Hydrogen Bonding and MM/GBSA Analyses of a Promising Compound in Complex with qmPfDHFR

**Figure 6:**
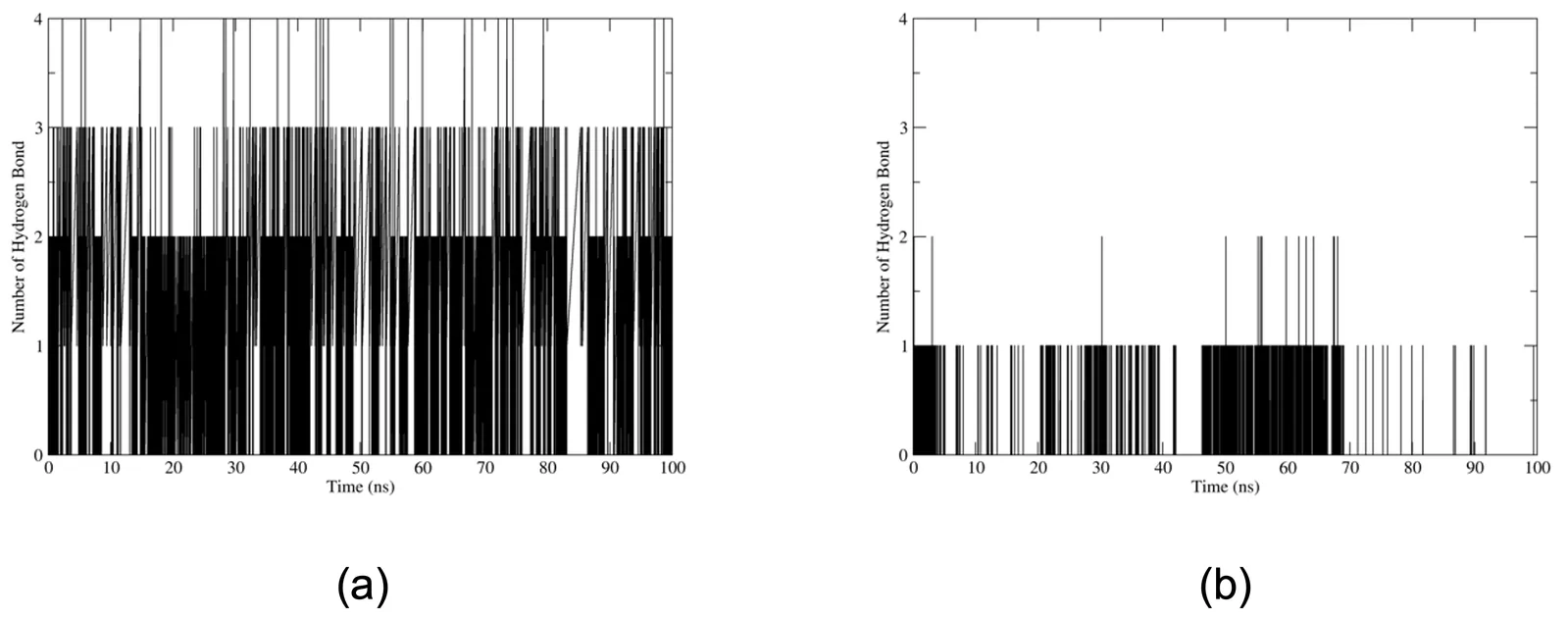
Hydrogen bond analysis of the qm*Pf*DHFR inhibitor WR99210 (**a**) and the promising compound glaucarubolone (**5**) (**b**) in complex with qm*Pf*DHFR over a 100 ns MD simulation.

To further assess the persistence and dynamic behavior of hydrogen bond interactions, hydrogen bond analysis was conducted over 100 ns of MD simulations. Hydrogen bonding between ligands and active-site residues contributes to ligand recognition and the stabilization of protein–ligand complexes. Analysis of the simulation trajectories demonstrated that all ligand–protein complexes maintained at least one hydrogen bond throughout the simulation period (Figure 6). WR99210 complexed with qm*Pf*DHFR exhibited an average of two hydrogen bond interactions throughout the simulation (Figure 6a). The most prominent interactions involved ASN108, with the highest individual hydrogen bond occupancy of 7.73%, followed by ALA16, which formed multiple parallel hydrogen bonds with different ligand atoms, resulting in a cumulative occupancy of approximately 8%. Additional contributions were observed for ILE14 (2.06%) and LEU164 (1.33%) (Supplementary Table S2). In contrast, glaucarubolone (**5**) complexed with qm*Pf*DHFR displayed a lower average number (approximately one hydrogen bond) throughout the simulation (Figure 6b). ASN108 was the predominant interacting residue, exhibiting the highest individual hydrogen bond occupancy of 0.8%, followed by ALA16 and LEU46, with occupancies of 0.10% and 0.05%, respectively (Supplementary Table S3). Although these hydrogen-bond interactions were transient and showed low occupancy throughout the simulation, the observed contacts with ALA16, LEU46, and ASN108 suggest that these residues may play a role in mediating transient interactions with glaucarubolone (**5**) and the qm*Pf*DHFR binding pocket.

In addition, the molecular mechanics/generalized Born surface area (MM/GBSA) approach was employed to estimate the binding free energy (BFE) of the reference inhibitor WR99210 and the promising compound glaucarubolone (**5**) in complex with qm*Pf*DHFR, based on equilibrated MD trajectories. This method integrates molecular mechanics energies with the generalized Born (GB) implicit solvent model and solvent-accessible surface area (SA) terms to evaluate the overall BFE. Individual energy components, including electrostatic, van der Waals, polar solvation, and non-polar solvation contributions for each ligand–protein complex, are summarized in Table 4. The MM/GBSA results indicate that both WR99210 and glaucarubolone (**5**) exhibit favorable binding to qm*Pf*DHFR, with BFE of −41.75 ± 3.41 and −36.53 ± 5.63 kcal/mol, respectively. The negative ΔG binding values indicate favorable predicted ligand–protein binding interactions. Notably, WR99210 demonstrated slightly stronger binding affinity than glaucarubolone (**5**), which is consistent with its role as a potent reference qm*Pf*DHFR inhibitor.

The electrostatic interaction energies (EEL) were negative for both systems, suggesting that Coulombic interactions contribute favorably to complex stability. WR99210 exhibited a more pronounced electrostatic contribution (−18.55 ± 4.21 kcal/mol) compared with glaucarubolone (**5**) (−8.17 ± 8.55 kcal/mol), indicating a greater contribution of polar or charged interactions in stabilizing the WR99210–qm*Pf*DHFR complex. By contrast, van der Waals interactions (VDW) were strongly negative for both ligands (−44.76 ± 2.90 kcal/mol for WR99210 and −41.52 ± 4.99 kcal/mol for glaucarubolone (**5**)), highlighting the dominant contribution of hydrophobic packing and dispersion forces in stabilizing both ligand binding within the active site of qm*Pf*DHFR. In contrast, polar solvation energies (ΔG_polar_) were positive (26.71 ± 3.11 kcal/mol for WR99210 and 17.72 ± 8.08 kcal/mol for glaucarubolone (**5**)), reflecting the energetic penalty associated with desolvation of polar functional groups upon binding, whereby polar or charged groups lose favorable interactions with the solvent. Conversely, non-polar solvation energies (ΔG_non-polar_) were negative (−5.15 ± 0.25 and −4.56 ± 0.39 kcal/mol for WR99210 and glaucarubolone (**5**), respectively), consistent with favorable hydrophobic contributions arising from the burial of solvent-accessible surface area during complex formation. Overall, these results suggest that ligand binding is primarily driven by VDW interactions with additional stabilization arising from EEL interactions, although these contributions are partially offset by unfavorable polar solvation effects.

Residue energy decomposition analysis of WR99210 in complex with qm*Pf*DHFR identified ILE14 and PHE58 as key hotspot residues contributing to ligand binding, with total energy contributions of −3.64 and −2.04 kcal/mol, respectively (Supplementary Table S4). ILE14 stabilized the ligand through a combination of VDW and EEL interactions, whereas PHE58 contributed predominantly through VDW interactions. In contrast, an analysis of the glaucarubolone (**5**)–qm*Pf*DHFR complex revealed PHE58 as a major stabilizing residue, contributing −2.10 kcal/mol, primarily through hydrophobic stacking interactions with the ligand (Supplementary Table S5). In addition, SER108 provides further stabilization (−1.09 kcal/mol) by reducing the electrostatic desolvation penalty, thereby enhancing the overall stability of the ligand–protein complex.

### 2.7. Drug-Likeness, Pharmacokinetic, and Toxicity Profiles of the Promising Compound

**Figure 7:**
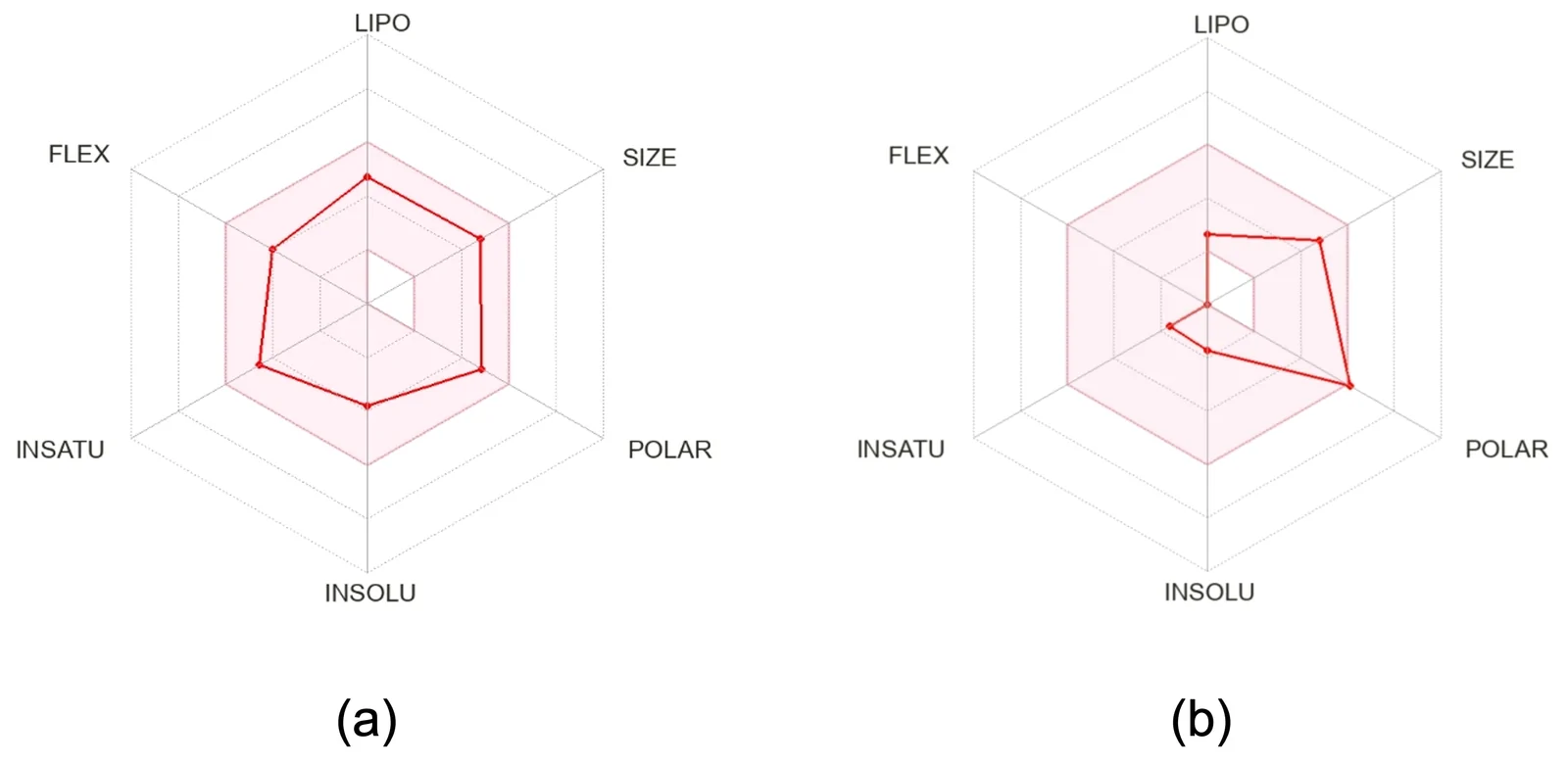
Bioavailability radar plots of compounds generated using the SwissADME web tool. The qm*Pf*DHFR inhibitor WR99210 (**a**) and the promising compound glaucarubolone (**5**) (**b**) are shown. Physicochemical parameters include lipophilicity (LIPO), molecular size (SIZE), polarity (POLAR), insolubility (INSOLU), insaturation (INSATU), and flexibility (FLEX). Red lines indicate the physicochemical profiles of respective compounds, while the pink region denotes the optimal ranges for drug-likeness.

Glaucarubolone (**5**) exhibited favorable binding interactions with qm*Pf*DHFR during MD simulations. To further assess the potential of glaucarubolone (**5**) as a drug candidate, its drug-likeness properties were evaluated to reduce the risk of failure in the drug development pipeline. Lipinski’s rule of five (Ro5) was applied, considering key physicochemical parameters, including molecular weight (MW ≤ 500 g/mol), number of hydrogen-bond donors (HBD ≤ 5), number of hydrogen-bond acceptors (HBA ≤ 10), and lipophilicity (CLogP ≤ 5), which are indicative of oral bioavailability [34]. Glaucarubolone (**5**) satisfied all Ro5 criteria (MW: 394.42 g/mol; HBD: 4; HBA: 8; CLogP: 0.07), suggesting favorable oral drug-like properties (Table 5). Similarly, WR99210 also complied with Ro5 (MW: 394.68 g/mol; HBD: 2; HBA: 4; CLogP: 2.68), consistent with its established inhibitory activity against qm*Pf*DHFR. In addition, bioavailability radar plot analysis was performed to further evaluate oral bioavailability based on six physicochemical parameters: lipophilicity (LIPO) (−0.7 < XLOGP3 < +5.0), size (150 < MW < 500 g/mol), polarity (POLAR) (20 Å^2^ < topological polar surface area (TPSA) < 130 Å^2^), insolubility (INSOLU) (−6 < LogS (ESOL) < 0), insaturation (INSATU) (0.25 < fraction Csp^3^ < 1), and flexibility (FLEX) (0 < rotatable bonds < 9) [35]. Glaucarubolone (**5**) met five of the six optimal criteria (XLOGP3 = −0.91; MW = 394.42 g/mol; LogS = −1.71; fraction Csp^3^ = 0.80; rotatable bonds = 0), with only polarity (TPSA = 133.52 Å^2^) slightly exceeding the optimal range (Figure 7b). In contrast, WR99210 satisfied all six parameters within the optimal range (XLOGP3 = 2.76; MW = 394.68 g/mol; TPSA = 98.46 Å^2^; LogS = −3.81; fraction Csp^3^ = 0.43; rotatable bonds = 6) (Figure 7a), supporting its predicted oral bioavailability.

In addition, the predicted pharmacokinetic properties and toxicity profiles of glaucarubolone (**5**) and WR99210 are presented in Table 6. With respect to absorption, glaucarubolone (**5**) was predicted to exhibit human intestinal absorption of 63.504%, whereas WR99210 showed a higher absorption rate of 80.022%. Both glaucarubolone (**5**) and WR99210 were predicted to be P-glycoprotein substrates but not P-glycoprotein inhibitors (P-gp I and II). In terms of distribution, glaucarubolone (**5**) exhibited low BBB permeability, with a Log BB value of −0.585, while WR99210 showed a higher BBB permeability value of −1.286 (Log BB < −1 indicating poor brain distribution). Regarding metabolism, glaucarubolone (**5**) was not predicted to be a substrate of either CYP2D6 or CYP3A4, whereas WR99210 was predicted to be a CYP3A4 substrate. Neither glaucarubolone (**5**) nor WR99210 was predicted to inhibit CYP1A2, CYP2C19, CYP2C9, CYP2D6, or CYP3A4. For excretion, glaucarubolone (**5**) had a higher predicted total clearance value (0.612 Log mL/min/kg) than WR99210 (0.387 Log mL/min/kg). Furthermore, glaucarubolone (**5**) was not predicted to be a renal OCT2 substrate, whereas WR99210 was predicted to be an OCT2 substrate. In terms of toxicity, glaucarubolone (**5**) exhibited a lower predicted toxicity profile than WR99210, with no predicted inhibition of hERG I or II and no predicted hepatotoxicity. In contrast, WR99210 was predicted to inhibit hERG II and exhibit hepatotoxicity. The predicted oral acute toxicity in rats was comparable between glaucarubolone (**5**) and WR99210, with values of 2.679 and 2.715 mol/kg, respectively. In contrast, glaucarubolone (**5**) showed a substantially higher predicted chronic oral toxicity threshold (3.375 Log mg/kg_bw/day) than WR99210 (1.383 Log mg/kg_bw/day), suggesting a potentially improved long-term safety profile.

## 3. Discussion

Malaria remains a major global health concern, and the efficacy of ACTs has been compromised by the emergence of partial artemisinin resistance in several endemic regions, including the Thailand–Myanmar border areas [2]. Medicinal plants have historically contributed to antimalarial drug discovery, including the development of quinine and artemisinin [6]. In this context, we investigated the antiplasmodial activity of EH, a species native to northeastern Thailand and Laos and closely related to EL [13,15,16]. Among the antiplasmodial activities of EL and EH root extracts, EL has been studied extensively. Previous studies have reported that the ethanolic and aqueous root extracts of EL exhibited in vitro antiplasmodial activity against the *P. falciparum* K1 strain, with IC_50_ values of 2.6 ± 0.8 μg/mL and >10 μg/mL, respectively [36]. However, no previous studies have reported the antiplasmodial activity of EH. To the best of our knowledge, the present study is the first to report its antiplasmodial potential. The EEHR (0.51 ± 0.10 μg/mL) exhibited slightly greater potency than the EELR (1.21 ± 1.03 μg/mL), which may be attributed to differences in their phytochemical compositions. Previous studies have reported that ethanolic extracts of EH contain significantly higher levels of total quassinoids (5.13 ± 0.21 mg/g dry weight), total canthin-6-one alkaloids (2.14 ± 0.13 mg/g dry weight), and total *β*-carboline alkaloids (6.20 ± 0.33 mg/g dry weight) compared with those of ethanolic extracts of EL across all three phytochemical classes [18]. Based on these previous findings, we postulate that the greater abundance of quassinoids in EH extracts may contribute, at least in part, to their enhanced antiplasmodial activity. However, the phytochemical composition of the EH extract was not comprehensively characterized in the present study. Therefore, further phytochemical profiling and quantitative analyses are warranted to determine whether the abundance of specific compound classes, particularly quassinoids, is associated with the observed antiplasmodial activity of EH extracts. Quassinoids are the principal bitter constituents of the Simaroubaceae family [37]. Their core skeletons are classified into five groups: C-18, C-19, C-20, C-22, and C-25 quassinoids [37]. In this study, two classes were identified: C-19 and C-20 quassinoids. The C-20 quassinoids exhibited greater antiplasmodial activity (IC_50_ = 0.13–0.27 μM) than the C-19 quassinoid (IC_50_ = 0.87 μM). Previous reports have demonstrated that eurycomalactone (IC_50_ = 0.21 μg/mL) displays antiplasmodial activity comparable to chloroquine (IC_50_ = 0.21 μg/mL) against the drug-resistant *P. falciparum* K1 strain [38]. This is consistent with the present findings, in which eurycomalactone (**3**) (IC_50_ = 0.87 μM) exhibited activity comparable to that of chloroquine (IC_50_ = 0.89 μM). The preliminary SAR analysis suggested that the presence of an oxymethylene bridge between C-8 and C-11 in ring C may contribute to the enhanced antiplasmodial potency observed among C-20 quassinoids compared with their C-19 analogs (Figure 3). This structural feature may be associated with enhanced antiplasmodial activity in C-20 quassinoids, whereas the C-19 analog eurycomalactone (**3**), which lacks this moiety, exhibits comparatively reduced activity. These findings are consistent with those of previous reports demonstrating that C-20 quassinoids, including eurycomanone, 13,21-dihydroeurycomanone, and 13α(21)-epoxyeurycomanone, exhibit higher activity against chloroquine-resistant *P. falciparum* (Gombak A strain) than the C-19 quassinoid, eurycomalactone [39]. Furthermore, C-20 quassinoids lacking an oxymethylene bridge, such as 11-dehydroklaineanone, 15β-hy-roxyklaineanone, 14,15β-dihydroxyklaineanone, and 15β-O-acetyl-14-hydroxyklaineanone, still display antiplasmodial activity against chloroquine-resistant *P. falciparum*, although with reduced potency (IC_50_ = 5.0–23.8 μM) compared to their bridged counterparts [40]. In addition, previous studies have suggested that the presence of an α,β-unsaturated ketone in ring A at C-2 is a key structural feature associated with enhanced antiplasmodial activity, as supported by comparisons with eurycomanol, which bears a hydroxyl group at C-2 [39]. In the present study, all quassinoids possessed an α,β-unsaturated ketone at C-2 of ring A (Figure 3), which likely contributed to their antiplasmodial effects. In addition, hydroxylation patterns play important roles in the modulation of biological activity. Previous studies have demonstrated that 14-hydroxychaparrinone, isolated from *Hannoa chlorantha* and *Hannoa klaineana*, exhibits higher IC_50_ values than chaparrinone, which lacks the C-14 hydroxyl group, against the *P. falciparum* NF54 strain [41]. Accordingly, compounds bearing a C-14 hydroxyl group in this study, such as eurycomanone (**1**), 13α(21)-epoxyeurycomanone (**2**), eurycomalactone (**3**), and glaucarubolone (**5**), appear to show only marginal differences in the antiplasmodial activity compared with chaparrinone (**4**), which lacks this substitution. In contrast, hydroxylation at C-15 in ring D may enhance the antiplasmodial activity (Figure 3). Glaucarubolone (**5**), which contains a hydroxyl group at C-15, exhibits greater potency than chaparrinone (**4**), which lacks this functional group. A similar trend was observed for eurycomanone (**1**) (IC_50_ = 0.14 ± 0.07 μM) and 13α(21)-epoxyeurycomanone (**2**) (IC_50_ = 0.16 ± 0.08 μM), both of which possess C-15 hydroxylation and display activities comparable to those of glaucarubolone (**5**) (IC_50_ = 0.13 ± 0.03 μM). In contrast, chaparrinone (**4**) exhibits slightly reduced activity (IC_50_ = 0.27 ± 0.03 μM). Consistent with these observations, previous studies have reported that chaparrinone isolated from *Castela texana* exhibits higher IC_50_ values (0.25 μg/mL) than glaucarubolone (0.12 μg/mL) against the *P. falciparum* D6 strain [42]. In addition, semisynthetic modification of the C-15 hydroxyl group of eurycomanone (IC_50_ = 0.56 μM) produced 15-*O*-isovaleryleurycomanone (IC_50_ = 0.65 μM), which retained comparable antiplasmodial activity against chloroquine-resistant *P. falciparum* Gombak A strain while exhibiting an eight-fold reduction in cytotoxicity toward brine shrimp relative to the parent compound [43]. Overall, based on these findings, we postulate that the C-15 position of C-20 quassinoids may play an important role in both antiplasmodial activity and cytotoxicity. However, given the limited number of compounds and structural analogs examined in the present study, further systematic medicinal chemistry and SAR investigations are warranted to determine whether modification at this position can improve antiplasmodial activity while reducing toxicity.

Among the alkaloid compounds, canthin-6-one (**6**) and its derivatives exhibited good to moderate antiplasmodial activity. The derivatives 9-methoxycanthin-6-one (**7**) (IC_50_ = 16.54 ± 1.16 μM) and canthin-6-one 9-O-*β*-glucopyranoside (**8**) (IC_50_ = 20.15 ± 4.75 µM) were more potent than the parent compound canthin-6-one (**6**) (IC_50_ = 47.31 ± 10.88 μM). Previous studies have reported that 5-methoxycanthin-6-one (5.1 μg/mL), isolated from *Zanthoxylum chiloperone*, exhibited antiplasmodial activity against the *P. falciparum* K1 strain and was slightly more potent than canthin-6-one (5.3 μg/mL) [44]. In the present study, a substitution at the C-9 position in ring A of the canthin-6-one scaffold may be associated with the observed differences in antiplasmodial activity (Figure 3). Nevertheless, further SAR studies are required to systematically evaluate the effects of different substitutions on the canthin-6-one core in relation to the antiplasmodial activity. In addition, the *β*-carboline alkaloids, including *β*-carboline-1-propionic acid (**9**), exhibit moderate antiplasmodial activity. This finding is consistent with earlier reports, in which 7-methoxy-*β*-carboline-1-propionic acid demonstrated weak activity [45]. Previous studies have also demonstrated that structural modification of the *β*-carboline scaffold, particularly at the C-1 and C-3 positions, can lead to improved and promising antiplasmodial activity [46]. Notably, cipargamin (KAE609), a *β*-carboline-based scaffold currently in phase II clinical trials, exhibits potent antiplasmodial activity [47]. In the present study, the quassinoids showed IC_50_ values ranging from 0.13 to 0.27 µM, making them approximately 130 to 271-fold more potent than *β*-carboline-1-propionic acid (**9**) (IC_50_ = 35.32 µM). This observation is consistent with previous reports demonstrating that eurycomanone is approximately 61 to 65-fold more active than *β*-carboline-1-propionic acid against *P. falciparum* D-6 and W-2 strains, respectively [45]. Nevertheless, these comparisons should be interpreted with caution, as differences in the *P. falciparum* strains and experimental conditions among studies may contribute to variations in the observed antiplasmodial activity.

Cytotoxicity assays revealed that eurycomalactone (**3**) exhibited the lowest CC_50_ value compared to the C-20 quassinoids. Previous studies have demonstrated that the C-19 quassinoid eurycomalactone possesses potent cytotoxic activity against different cancer cell lines, with IC_50_ values ranging from 0.59 to 0.78 μM [48]. This activity is comparable to that of the anticancer agent doxorubicin (IC_50_ = 0.66–0.86 μM) and greater than that of eurycomanone-type quassinoids, such as 13,21-dihydroeurycomanone (IC_50_ = 5.8–100 μM) [48]. The cytotoxicity of eurycomalactone has been attributed to several key structural features, including keto groups at C-2 and C-7, a double bond between C-3 and C-4, and the absence of substitutions at C-5 and C-6 [48]. In addition, eurycomalactone has been reported to inhibit protein synthesis in immortalized human umbilical vein endothelial cells (HUVECtert) [49]. Consistent with these findings, the present study demonstrated that eurycomalactone (**3**) exhibited higher cytotoxicity toward normal mammalian Vero cells (CC_50_ = 5.97 μM) than the C-20 quassinoids (CC_50_ = 6.11–28.84 μM). However, the CC_50_ value of eurycomalactone (**3**) in Vero cells was higher than that previously reported for cancer cell lines, indicating comparatively lower sensitivity in normal cells. Therefore, further evaluations using additional human cell lines are required. In contrast, all alkaloid compounds exhibited higher CC_50_ values, ranging from 21.95 to >416 μM, indicating lower cytotoxicity than quassinoids. None of the EL or EH root extracts exhibited cytotoxicity toward Vero cells, with CC_50_ values exceeding 30 μg/mL [50].

The present study showed that EL and EH root extracts, as well as eurycomanone (**1**) and glaucarubolone (**5**), affected the erythrocytic development of *P. falciparum* K1 strain, as indicated by morphological alterations. Similar effects were observed with artesunate. Notably, host erythrocytes remained structurally intact, whereas morphological changes were confined to intracellular parasites after treatment with extracts and compounds derived from EL and EH roots. These findings are consistent with those of previous studies on *Brucea javanica* (L.) (Simaroubaceae), in which both root and fruit extracts induced morphological alterations in *P. falciparum* NF54, particularly at the ring stage, through nuclear clumping, leading to pyknotic cell death [51]. This effect was attributed to the major quassinoid constituents of *B. javanica* [51]. Furthermore, the present results agree with earlier reports on standard antimalarial drugs, including artesunate and chloroquine. Artesunate-treated *P. falciparum* cultures exhibited rapid parasite killing, characterized by the appearance of pyknotic nuclei within 24 h, reflecting its fast-acting mechanism of action [52]. Similarly, artesunate exposure of *P. falciparum* isolates from Thai patients resulted in arrested parasite development, with parasites appearing small, shrunken, and pyknotic within 1–2 h [53]. In contrast, chloroquine treatment of *P. falciparum* K1-resistant strains, even at concentrations up to 10-fold higher than IC_50_, did not completely inhibit parasite development [54]. Consistently, parasites treated with chloroquine in the present study were able to complete their developmental cycle and reinvade new erythrocytes after 48 h of incubation. These observations are consistent with the in vitro antiplasmodial activity results, as chloroquine exhibited a higher IC_50_ value than eurycomanone (**1**) and glaucarubolone (**5**), reflecting partial parasite survival and the ability of a substantial proportion of parasites to progress to the second infection cycle through the re-invasion of new host erythrocytes. In the case of quassinoids, eurycomanone has been reported to exert potent activity against the ring stage and inhibit the progression of young trophozoites to mature schizonts in chloroquine-resistant *P. falciparum* FCR-3 within 24 h of in vitro incubation [55]. Consistent with these findings, our observations of eurycomanone (**1**) and glaucarubolone (**5**) further support the potential of quassinoid compounds as fast-acting antiplasmodial agents targeting early intraerythrocytic stages.

Based on these observations, one possible mechanism underlying the inhibition of parasite development by EL and EH root extracts may be primarily attributed to their major bioactive quassinoid constituents, particularly eurycomanone (**1**) and glaucarubolone (**5**), which exhibited excellent in vitro antiplasmodial activity and induced marked alterations in parasite development. Previous studies have suggested that quassinoids act as rapid and potent inhibitors of protein synthesis, with subsequent downstream effects on protein and nucleic acid synthesis in *P. falciparum* [56]. Notably, the progression of the parasite from the ring stage to the mature schizont requires continuous synthesis of essential proteins at each developmental stage. Therefore, quassinoids present in the extracts, including eurycomanone (**1**) and glaucarubolone (**5**), may disrupt this process, ultimately leading to the failure of maturation into schizonts. Alkaloid compounds were not selected for morphological evaluation in the present study because of their comparatively limited antiplasmodial activity. Previous reports have suggested that compounds containing a *β*-carboline scaffold may inhibit parasite growth by interfering with DNA synthesis, potentially through intercalation between DNA base pairs [57]. Accordingly, it is plausible that the combined presence of quassinoids, *β*-carboline alkaloids, canthin-6-one alkaloids, and other unidentified constituents may contribute to the observed antiplasmodial activity through multiple mechanisms of action. The distinct morphological alterations observed in the extract-treated parasites, compared to those exposed to individual compounds, may reflect synergistic interactions among these constituents. Further investigation using transmission electron microscopy is warranted to elucidate ultrastructural changes at a higher resolution, particularly with respect to specific organelles and structural damage induced by EL and EH root extracts and their derived compounds. Moreover, the precise mechanisms underlying the antiplasmodial activities of these compounds, particularly glaucarubolone (**5**), remain to be fully elucidated and warrant further investigation.

To gain insight into the potential molecular basis of the observed antiplasmodial activity, molecular docking was performed to explore the interactions of the promising compounds with qm*Pf*DHFR. In this study, RMSD obtained for WR99210 (0.608 Å) was lower than previously reported values of 0.971–1.518 Å [58], indicating that the docking protocol used in the present study provided a comparable or improved reproduction of the crystallographic binding conformations. Furthermore, the docking results agreed with those of previous studies, demonstrating that WR99210 forms hydrogen bonds with residues, such as ILE14 and CYS15, along with multiple hydrophobic interactions within the active site [59]. In addition, ASP54 plays a crucial role in the catalytic mechanism of *Pf*DHFR by facilitating hydride transfer from the NADPH cofactor to DHF, thereby generating THF [21,59]. The inhibition of this process by antifolate compounds, including pyrimethamine, cycloguanil, and WR99210, disrupts downstream folate metabolism and impairs parasite growth and proliferation [59]. Although the NADPH cofactor was not retained in the docking and MD simulations, the observed interactions with ASP54 and ASN108 suggest that the quassinoids may engage functionally important regions of the qm*Pf*DHFR active site. These interactions may influence the local structural environment of the binding site and potentially affect substrate or cofactor recognition. However, because NADPH was not included in the present simulations, the extent to which these interactions affect NADPH binding or cofactor-mediated catalytic activity cannot be determined from the current computational results alone. Accordingly, further biochemical and mechanistic studies are warranted to elucidate the functional significance of these interactions. Similar interaction patterns were observed for canthin-6-one and *β*-carboline alkaloids, although they had lower binding affinities than quassinoids. A previous study reported that the C-20 quassinoid soulameanone, isolated from *Brucea mollis*, exhibited favorable binding interactions with qm*Pf*DHFR (binding energy = −8.78 kcal/mol), forming hydrogen bonds with key active-site residues, including ALA16, ASN108, SER111, LEU164, and TYR170 [60]. In contrast, the C-20 quassinoids identified in this study displayed a distinct binding pattern, with prominent hydrogen bonding to ASP54, ASN108, and SER111. This difference may be attributed to the absence of an oxymethylene bridge between C-8 and C-11 in ring C of soulameanone, which may influence the ligand orientation within the qm*Pf*DHFR active site [60]. Among the alkaloid compounds, 9-methoxycanthin-6-one exhibited a weaker binding affinity (−6.25 kcal/mol) than soulameanone (−8.78 kcal/mol) [60]. Similarly, in the present study, canthin-6-one alkaloids (canthin-6-one (**6**) and 9-methoxycanthin-6-one (**7**)) demonstrated binding energies of approximately −6 kcal/mol, whereas quassinoid compounds demonstrated stronger interactions, with binding energies ranging from −8 to −9 kcal/mol. Although relatively few studies have investigated natural compounds targeting qm*Pf*DHFR, computational analyses have demonstrated that several alkaloids from *Cryptolepis sanguinolenta* exhibit favorable binding, particularly through hydrogen bonding, with the critical ASN108 residue [59]. Furthermore, the present findings, which indicate more favorable interactions of artesunate than chloroquine with qm*Pf*DHFR, are consistent with those of previous reports. Artemisinin has been reported to demonstrate more favorable binding than chloroquine in the qm*Pf*DHFR active site using AutoDock Vina [61]. Overall, these findings suggest that quassinoids may represent potential antimalarial scaffolds with plausible interactions with qm*Pf*DHFR, as indicated by favorable in silico binding profiles, warranting further experimental validation using enzymatic assays.

To further investigate the structural dynamics and conformational behavior of qm*Pf*DHFR in complexes with glaucarubolone (**5**) and WR99210, as well as the ligand-free (apo) qm*Pf*DHFR, RMSD, RMSF, and RoG were evaluated throughout the 100 ns MD simulations [62]. The RMSD profiles indicated that the glaucarubolone (**5**)–qm*Pf*DHFR complex exhibited relatively stable conformational behavior throughout the simulation, with a mean RMSD comparable to that of the WR99210–qm*Pf*DHFR complex. In contrast, apo qm*Pf*DHFR showed a higher mean RMSD than both ligand-bound systems, suggesting greater overall structural deviation during the simulation. The RMSD profiles of both ligand-bound systems remained relatively stable throughout the production simulations without substantial systematic drift over time, suggesting that both complexes maintained relatively stable overall conformations during the simulations [31]. For the ligands, glaucarubolone (**5**) exhibited a slightly higher mean RMSD than WR99210, indicating greater positional fluctuations during the simulation. This behavior may reflect minor positional or conformational adjustments of glaucarubolone (**5**) within the qm*Pf*DHFR binding site. The RMSF analysis showed that the glaucarubolone (**5**)–qm*Pf*DHFR complex exhibited slightly lower residue fluctuations compared with the WR99210-bound and apo qm*Pf*DHFR systems. Notably, ASN108 displayed markedly lower RMSF in the glaucarubolone (**5**)–qm*Pf*DHFR complex, indicating reduced local flexibility of this residue in the ligand-bound system. This observation is consistent with the hydrogen-bond interaction formed between glaucarubolone (**5**) and ASN108, which may contribute to restricting local residue mobility within the binding site. Furthermore, ASN108 was identified as one of the key residues contributing favorably to the binding energy in the MM/GBSA energy decomposition analysis. The combination of reduced RMSF, hydrogen-bond formation, and favorable energy contribution suggests that ASN108 may play an important role in the interaction of glaucarubolone (**5**) with qm*Pf*DHFR and may contribute to maintaining its ligand binding within the active site. Despite the lower RMSF observed for the glaucarubolone (**5**)-bound system, the mean RMSF values were relatively comparable among the three systems, consistent with previous findings that ligand binding to *Pf*DHFR results in minimal structural deviations and only minor conformational changes in the protein [62]. In addition, although differences in the mean RoG values were observed among the glaucarubolone (**5**)–qm*Pf*DHFR, WR99210–qm*Pf*DHFR, and apo qm*Pf*DHFR systems, the overall variation in RoG remained relatively small. This suggests that ligand binding was not associated with substantial changes in the global compactness of qm*Pf*DHFR during the simulations. This finding is consistent with previous studies reporting only minor changes in the RoG of *Pf*DHFR following ligand binding [30,31].

Moreover, hydrogen bond analysis demonstrated that both glaucarubolone (**5**) and WR99210 formed hydrogen bond interactions with qm*Pf*DHFR during the MD simulations. WR99210 exhibited higher hydrogen-bond occupancy than glaucarubolone (**5**), indicating that its hydrogen-bond interactions were relatively more persistent throughout the simulations. Previous studies have demonstrated that WR99210 can accommodate active site mutations, including the substitution of SER108 with ASN108, while maintaining favorable binding interactions [63]. In addition, consistent with this observation, WR99210 exhibited a slightly lower mean ligand RMSD than glaucarubolone (**5**), suggesting reduced positional fluctuations during the simulation. In contrast, the slightly higher ligand RMSD and lower hydrogen-bond occupancy observed for glaucarubolone (**5**) suggest greater positional variation and less persistent hydrogen-bond interactions during the simulation. The absence of rotatable bonds in glaucarubolone (**5**), compared with six rotatable bonds in WR99210, may limit its ability to adapt to positional changes within the active site and consequently reduce the persistence of its hydrogen-bond interactions (Table 5) [30].

A comparative evaluation of BFE provided insights into predicted binding characteristics of the investigated compounds relative to the reference inhibitor WR99210 [30]. Previous studies have demonstrated that WR99210 complexed with wild-type (wt) *Pf*DHFR is predominantly stabilized by van der Waals interactions, as determined using the MM/GBSA approach [30]. Furthermore, molecular mechanics Poisson–Boltzmann surface area (MM/PBSA) analyses have demonstrated that the BFE of WR99210 with both wt*Pf*DHFR and qm*Pf*DHFR is comparable, with van der Waals interactions, followed by electrostatic interactions, serving as the dominant stabilizing forces [64]. Per-residue free-energy decomposition analyses identified ILE14 and PHE58 as the principal contributors to WR99210 binding [64]. Consistent with these reports, our results indicated that WR99210 binding is primarily driven by interactions with key residues, particularly ILE14 and PHE58, within the qm*Pf*DHFR active site. In comparison, the promising compound glaucarubolone (**5**) in this study exhibited a slightly less favorable BFE than WR99210, suggesting a weaker predicted binding affinity towards qm*Pf*DHFR. Although hydrogen bond analysis showed that the hydrogen bond interactions of glaucarubolone (**5**) were predominantly transient, its favorable BFE suggests that other non-hydrogen-bond interactions may contribute substantially to its favorable predicted binding within the binding pocket. In particular, the favorable van der Waals, electrostatic, and non-polar energy contributions may contribute to the overall favorable binding energy of glaucarubolone (**5**) and appear to play important roles in stabilizing the glaucarubolone (**5**)-qm*Pf*DHFR complex. Furthermore, consistent with this observation, per-residue free-energy decomposition analysis indicated PHE58 and ASN108 as key residues contributing favorably to the predicted binding interaction of glaucarubolone (**5**) to qm*Pf*DHFR. Interestingly, the initial docking score and the final MM/GBSA BFE exhibited different rankings for WR99210 and glaucarubolone (**5**). This discrepancy highlights the inherent limitations of static docking approaches, which use a single or limited receptor conformation and simplified empirical scoring functions that may not fully account for receptor flexibility and solvation effects. Previous studies have similarly reported that the use of empirical scoring functions and heuristic search algorithms can contribute to computational limitations and inherent inaccuracies in molecular docking predictions [65]. In contrast, the MD-coupled MM/GBSA method evaluates binding over a dynamic ensemble of conformations, allowing conformational adaptations of the receptor and ligand during the simulations and providing a dynamic perspective on ligand–protein interactions. Additionally, the MM/GBSA approach incorporates an implicit solvation model (GB/SA), accounting for both polar and non-polar solvation contributions that can influence the predicted BFE. Consequently, MM/GBSA analysis provides a complementary perspective on predicted binding energetics, supporting the more favorable predicted binding of WR99210 compared with glaucarubolone (**5**) under dynamic and solvated conditions. Overall, the combined molecular docking, MD, hydrogen-bond, and MM/GBSA analyses suggest that glaucarubolone (**5**) can maintain favorable predicted interactions with qm*Pf*DHFR under the simulated conditions, although these interactions appeared less persistent than those observed for WR99210. Nevertheless, further in vitro enzymatic assays are required to determine whether glaucarubolone (**5**) directly inhibits qm*Pf*DHFR and to experimentally validate the predicted binding interactions. Additionally, medicinal chemistry-driven structural optimization together with systematic evaluation of structurally related analogues may provide additional insights into the SAR of glaucarubolone (**5**) and facilitate the development of more potent and selective antiplasmodial derivatives.

Oral administration of antimalarial drug therapy is the preferred route because it improves patient compliance, facilitates drug administration, and reduces medical and healthcare costs [66]. In addition, the oral route is particularly beneficial in resource-limited settings, where access to healthcare facilities and specialized medical personnel may be restricted. Currently, ACTs represent the first-line treatment for uncomplicated *P. falciparum* malaria and are administered orally. In contrast, severe malaria requires parenteral administration of artemisinin derivatives to achieve rapid reductions in parasitemia. Therefore, the early prediction of oral bioavailability based on physicochemical properties provides a valuable preliminary approach for assessing the suitability of potential antimalarial candidates for oral therapy. Drug-likeness predictions indicated that glaucarubolone (**5**) complies with Ro5 and exhibits physicochemical characteristics consistent with favorable drug-like properties. Furthermore, it fulfilled five of the six bioavailability radar parameters, suggesting potential oral suitability despite its slightly elevated polarity. In comparison, the qm*Pf*DHFR inhibitor WR99210 served as a reference and satisfied all evaluated physicochemical parameters, supporting its use as a benchmark compound. Beyond drug-likeness properties, pharmacokinetic and toxicity predictions provide important preliminary information for assessing drug candidates during early stages of drug development and may help reduce the risk of failure in clinical trials [67]. Glaucarubolone (**5**) showed generally favorable predicted pharmacokinetic properties but demonstrated moderate intestinal absorption compared with WR99210. This finding is consistent with previous studies reporting moderate absorption and limited bioavailability of quassinoids in animal models [68,69]. Glaucarubolone (**5**) also showed low predicted BBB permeability, which may be advantageous in uncomplicated *P. falciparum* malaria by limiting CNS exposure but could be a limitation in cerebral malaria, where BBB penetration could be necessary to achieve therapeutic efficacy. The moderate intestinal absorption and low BBB permeability of glaucarubolone (**5**) may be attributable, at least in part, to its physicochemical properties, particularly its relatively high TPSA, which was outside the optimal range indicated in the bioavailability radar plot. Because TPSA is an important determinant of passive membrane permeability, this property may contribute to the limited intestinal absorption and BBB permeability of glaucarubolone (**5**) [70]. In addition, glaucarubolone (**5**) was not predicted to inhibit the investigated or any of the CYP isoforms, suggesting a low potential for CYP-mediated drug–drug interactions (DDIs), similar to WR99210. Furthermore, its higher predicted clearance may also reduce the risk of drug accumulation during prolonged exposure. Regarding toxicity, glaucarubolone (**5**) showed no predicted hERG I/II inhibition or hepatotoxicity, indicating a potentially lower risk of cardiotoxicity and hepatotoxicity than WR99210, which was predicted to inhibit hERG II and exhibit hepatotoxicity. Notably, WR99210 has previously been discontinued from clinical development due to toxicity-related concerns, while its derivative P218 was subsequently developed to improve its safety profile [71]. In addition, previous studies have reported that quassinoids generally exhibit mild cytotoxicity towards normal human cells and do not induce apparent organ toxicity in mice [72]. Overall, these findings suggest that glaucarubolone (**5**) demonstrated favorable predicted drug-likeness and pharmacokinetic properties, alongside a comparatively low predicted toxicity profile. Nevertheless, these in silico findings require confirmation through appropriate in vivo studies, and further medicinal chemistry structural optimization may help enhance its pharmacokinetic properties and overall safety profiles.

## 4. Materials and Methods

### 4.1. Plant Samples and Preparation

EL and EH roots were collected from the Yala and Ubon Ratchathani provinces of Thailand, respectively. The plant materials were obtained from cultivated fields managed by local farmers. All plant collections were conducted in accordance with national regulations and guidelines for plant material collection. Plant materials were taxonomically authenticated by Dr. Gorawit Yusakul (Faculty of Pharmaceutical Sciences, Naresuan University, Thailand) and Assistant Professor Dr. Thaweesak Juengwatanatrakul (Faculty of Pharmaceutical Sciences, Ubon Ratchathani University, Thailand). Voucher specimens (EL: PHWU53 and EH: PHWU54) were deposited at the School of Medicine, Walailak University, Thailand. The collected roots were thoroughly washed to remove adhering soil and impurities and subsequently dried in a hot air oven at 60 °C for 72 h. Dried samples were ground into a fine powder and stored in airtight containers before extraction. Extractions were performed according to a previously reported protocol [18]. Briefly, 50 mg of the powdered sample was transferred into a 1.5 mL microcentrifuge tube and extracted separately with 1 mL of either 50% (*v*/*v*) ethanol or boiled distilled water. Ultrasonication was applied for 20 min to facilitate the extraction. Next, the mixtures were centrifuged at 4300× *g* for 10 min, and the supernatants were carefully collected. The extraction process was repeated four times for each sample to ensure maximum yield, and all supernatants were pooled. The ethanolic extracts were evaporated using a rotary evaporator, and the aqueous extracts were freeze-dried (lyophilized). The resulting plant extracts were stored in sealed containers at 4 °C until further use.

### 4.2. Chemicals

C-20 quassinoid compounds, including eurycomanone (**1**) (C_20_H_24_O_9_; 98% purity), 13α(21)-epoxyeurycomanone (**2**) (C_20_H_24_O_10_; 98% purity), and the C-19 quassinoid eurycomalactone (**3**) (C_19_H_24_O_6_; 98% purity), were purchased from Chengdu Biopurify Phytochemicals (Chengdu, China). Additional authentic C-20 quassinoid compounds, including chaparrinone (**4**) (C_20_H_26_O_7_; 98% purity) and glaucarubolone (**5**) (C_20_H_26_O_8_; 98% purity), as well as canthin-6-one alkaloids, including canthin-6-one (**6**) (C_14_H_8_N_2_O; 98% purity), 9-methoxycanthin-6-one (**7**) (C_15_H_10_N_2_O_2_; 96% purity), and canthin-6-one 9-O-*β*-glucopyranoside (**8**) (C_20_H_18_N_2_O_7_; 97% purity), and the *β*-carboline alkaloid *β*-carboline-1-propionic acid (**9**) (C_14_H_12_N_2_O_2_; 99% purity), were obtained as authentic standards. These authentic compounds were previously isolated from the roots of EH by our research group and structurally characterized using nuclear magnetic resonance (NMR) spectroscopy and mass spectrometry, as reported previously [18,19,20]. Furthermore, HPLC compositional analysis showed that the EH root extract contained higher levels of quassinoids (5.13 mg/g dry weight), canthin-6-one alkaloids (2.14 mg/g dry weight), and *β*-carboline alkaloids (6.20 mg/g dry weight), representing the major phytochemical classes in the extract to which the authentic compounds described above belong. In contrast, the EL root extract contained lower levels of these phytochemical classes (1.68, 0.88, and 1.31 mg/g dry weight, respectively), as previously reported [18]. The two-dimensional chemical structures of these compounds (Figure 3) were generated using MarvinSketch v.22.12.0.

The reference antimalarial drugs, artesunate (C_19_H_28_O_8_; 99% purity) and chloroquine diphosphate salt (C_18_H_32_ClN_3_O_8_P_2_; 98.5% purity), were purchased from Sigma-Aldrich (St. Louis, MO, USA) and used as positive controls in the antiplasmodial assays. Doxorubicin hydrochloride (C_27_H_30_ClNO_11_, 98% purity) was purchased from Sigma-Aldrich (St. Louis, MO, USA) and used as a positive control in the cytotoxicity assays. All plant extracts were dissolved in distilled water or dimethyl sulfoxide (DMSO) (Merck, Boston, MA, USA), depending on the extraction solvent. The pure compounds were dissolved in DMSO. The stock solutions were further diluted with culture medium to obtain a final DMSO concentration of 0.1% (*v*/*v*).

### 4.3. Parasites and Cell Line

The drug-resistant *P. falciparum* K1 strain was obtained from Dr. Rapatbhorn Patrapuvich of the Faculty of Tropical Medicine, Mahidol University, Thailand. The parasites were cultured and maintained to evaluate the antiplasmodial activity according to an established protocol [73]. Vero cells (ATCC CCL-81) were used as mammalian cells for cytotoxicity testing and were cultured and maintained under standard conditions following an established protocol [73].

### 4.4. In Vitro Antiplasmodial Activity

The antiplasmodial activities of plant extracts and compounds derived from EL and EH roots were evaluated using a parasite lactate dehydrogenase (pLDH) assay, which measures pLDH activity in viable parasites, as previously described by Makler and Hinrichs [74].

Briefly, *P. falciparum* K1 strain-infected erythrocytes were diluted with uninfected erythrocytes in Roswell Park Memorial Institute (RPMI) 1640 medium (Gibco, Grand Island, NY, USA) to obtain a final suspension containing 2% parasitemia and 2% hematocrit. A volume of 180 μL of parasite suspension was dispensed into each well of a 96-well microplate. All EL and EH extracts, their derived compounds, and reference antimalarial drugs were serially diluted two-fold from their respective stock solutions. Subsequently, 20 μL of each test sample was added to the wells to obtain the designated final concentrations. Each concentration was tested in triplicate, and the assay was performed in three independent experiments. The aqueous and ethanolic extracts of EL roots (AELR and EELR) and EH roots (AEHR and EEHR) were evaluated at final concentrations ranging from 50 to 0.39 μg/mL. Quassinoid compounds (**1**)–(**5**) were evaluated at concentrations of 3.12 to 0.02 μg/mL, whereas alkaloid compounds (**6**)–(**9**) were tested at 100 to 0.78 μg/mL. Artesunate and chloroquine were used as positive controls at concentrations ranging from 20 to 0.002 μg/mL. Wells treated with 0.1% DMSO served as negative controls, whereas uninfected erythrocytes served as baseline controls. Plates were incubated at 37 °C in a 5% CO_2_ incubator for 72 h. Following incubation, erythrocytes were lysed by three freeze–thaw cycles (−20 °C for 30 min and 37 °C for 30 min). Subsequently, 100 μL of Malstat reagent and 20 μL of nitroblue tetrazolium/phenazine ethosulfate solution were added to each well of a new 96-well plate, followed by the transfer of 20 μL of the lysed samples. The plates were incubated in the dark at room temperature for 1 h. The absorbance was measured at 650 nm using a Multiskan SkyHigh microplate spectrophotometer (Thermo Fisher Scientific, Waltham, MA, USA), as per the previously reported protocol [73]. The percentage inhibition of parasite growth was calculated according to Equation (1), and the half-maximal inhibitory concentration (IC_50_) values were determined using GraphPad Prism v9 (GraphPad Software, La Jolla, CA, USA).

(1) %inhibition = (A − B/A) × 100

A = Absorbance of negative control;
B = Absorbance of tested samples.

### 4.5. In Vitro Cytotoxicity

The objective of this study was to evaluate the cytotoxic effects of plant extracts and compounds derived from EL and EH roots. This assay is based on the ability of metabolically active cells to reduce the water-soluble yellow dye 3-(4,5-dimethylthiazol-2-yl)-2,5-diphenyltetrazolium bromide (MTT) into insoluble purple formazan crystals, a process that occurs in cells with intact mitochondrial function. The MTT assay was performed according to an established protocol [73].

Briefly, Vero cells were dissociated into single-cell suspensions using 3 mL of 0.05% trypsin–EDTA solution (Gibco, Grand Island, NY, USA) and incubated at 37 °C in a 5% CO_2_ incubator for 5 min. The cell suspension was centrifuged at 2500 rpm for 5 min, and the supernatant was discarded. The resulting cell pellet was resuspended in Dulbecco’s Modified Eagle Medium (DMEM) (HyClone, Singapore), and the cells were counted using a hemocytometer (Boeco, Hamburg, Germany). Cells were seeded into 96-well culture plates at a density of 1 × 10^4^ cells per well in a final volume of 180 μL and incubated at 37 °C in a 5% CO_2_ incubator for 24 h to allow cell attachment. Following incubation, EL and EH root extracts and their derived compounds (**1**)–(**9**) were added to the respective wells by adding 20 μL of each test sample to achieve final concentrations ranging from 100 to 0.78 μg/mL. Doxorubicin was included as a positive control at final concentrations ranging from 10 to 0.07 μg/mL, while cells treated with 0.1% DMSO served as the negative control. Each concentration was tested in triplicate, and the assay was performed in three independent experiments. The plates were further incubated at 37 °C in a 5% CO_2_ incubator for 48 h. Subsequently, the culture supernatant was carefully removed, and 50 μL of MTT solution (5 mg/mL) was added to each well. After incubation for an additional 2 h under the same conditions, the MTT solution was discarded, and 100 μL of DMSO was added to dissolve the formazan crystals. The absorbance was measured at 560 nm with background subtraction at 670 nm using a Multiskan SkyHigh microplate spectrophotometer (Thermo Fisher Scientific, Waltham, MA, USA). The absorbance values were used to calculate the percentage of cytotoxicity according to Equation (2). The 50% cytotoxic concentration (CC_50_) was determined using the GraphPad Prism v9 (GraphPad Software, La Jolla, CA, USA).

(2) %cytotoxicity = 100 − ((A − B)/(C − B) × 100)

A = Absorbance of tested samples;
B = Absorbance of background;
C = Absorbance of negative control.

### 4.6. Selectivity Index (SI) Determination

The SI was used to identify plant extracts and compounds derived from EL and EH roots that exhibit potential for further investigation. The SI values were calculated as the ratio of the 50% cytotoxic concentration (CC_50_) in Vero cells to the half-maximal inhibitory concentration (IC_50_) against the *P. falciparum* K1 strain according to Equation (3). A higher SI value indicates greater selectivity of an extract or compound toward the parasite relative to normal cells. Extracts or compounds with SI values greater than 10 were considered promising candidates for further investigation [29].

(3) Selectivity index (SI) = A/B

A = The 50% cytotoxic concentration (CC_50_) against Vero cells;
B = The half maximal inhibitory concentration (IC_50_) against the *P. falciparum* K1 strain.

### 4.7. Parasite Morphological Changes Following Exposure to Plant Extracts and Promising Compounds

The effects of plant extracts and compounds derived from the EL and EH roots on parasitic morphology were evaluated. Briefly, *P. falciparum* K1 strain parasites were synchronized using 5% D-sorbitol to obtain ring-stage parasites prior to the initiation of the experiments, following a previous protocol [75,76]. The synchronized cultures were then adjusted to 2% parasitemia and 2% hematocrit and exposed to the respective IC_90_ concentrations of plant extracts and selected promising compounds. Artesunate and chloroquine were used as positive controls, whereas parasite cultures treated with 0.1% DMSO served as negative controls, following a previously described protocol [77]. The treated cultures were incubated at 37 °C in a 5% CO_2_ incubator for 72 h. Giemsa-stained thin blood smears were prepared at 0 (baseline), 6, 12, 24, 36, 48, and 60 h post-treatment. The smears were stained with Wright–Giemsa stain solution (Biotech Reagent, Bangkok, Thailand) for 5 min, followed by immersion in buffer solution for an additional 5 min. Parasite morphology was examined under a light microscope (Olympus CX31; Tokyo, Japan) using a 100× oil immersion objective. The morphological classification of intraerythrocytic blood-stage parasites and their representative abnormal forms was performed according to a previously reported study [78]. The predominant morphological changes observed at each time point were recorded and are presented as representative findings for each treatment condition.

### 4.8. Molecular Docking Simulations

Molecular docking was performed to investigate the interactions between the compounds derived from EL and EH roots and the quadruple mutants (N51I, C59R, S108N, and I164L) of *P. falciparum* dihydrofolate reductase (qm*Pf*DHFR).

The three-dimensional (3D) structure of qm*Pf*DHFR (PDB ID: 1J3K), complexed with the potent inhibitor WR99210 [6,6-dimethyl-1-(3-(2,4,5-trichlorophenoxy)propoxy)-1,6-dihydro-1,3,5-triazine-2,4-diamine], and resolved by X-ray crystallography at a resolution of 2.10 Å, was retrieved from the RCSB Protein Data Bank (RCSB PDB) (https://www.rcsb.org/) [63] (accessed on 11 December 2025). Missing residues were modeled into the qm*Pf*DHFR protein structure. The protein structure was prepared using the PDB2PQR web server by assigning atomic charges and radii based on the AMBER force field [79,80]. The NADPH cofactor and water molecules were subsequently removed following the protocol adopted in previous studies [30,31]. Protonation states and ionizable residues were determined using PROPKA3 by simulation at pH 7.15, reflecting the cytoplasmic environment of the parasite [79,80]. The structure was further refined using MolProbity for atom contact correction and hydrogen atom addition, thereby improving structure quality [79,81]. Subsequently, Kollman charges were added using AutoDockTools v1.5.6 [82].

The 3D structures of ligands were retrieved from the PubChem database (https://pubchem.ncbi.nlm.nih.gov/) (accessed on 11 December 2025), including eurycomanone (**1**) (PubChem CID: 13936691), 13α(21)-epoxyeurycomanone (**2**) (PubChem CID: 13936703), eurycomalactone (**3**) (PubChem CID: 441793), chaparrinone (**4**) (PubChem CID: 73154), glaucarubolone (**5**) (PubChem CID: 441797), canthin-6-one (**6**) (PubChem CID: 97176), 9-methoxycanthin-6-one (**7**) (PubChem CID: 9881423), canthin-6-one 9-O-*β*-glucopyranoside (**8**) (PubChem CID: 637482), and *β*-carboline-1-propionic acid (**9**) (PubChem CID: 5375436). In addition, the reference antimalarial drugs artesunate (PubChem CID: 6917864) and chloroquine (PubChem CID: 2719) were included for comparison. Gasteiger partial charges were assigned to all ligand structures using AutoDockTools v1.5.6 [82], followed by geometry optimization using the Universal Force Field (UFF) implemented in Open Babel v2.4.1 [83]. Both the prepared protein and ligand structures were saved in PDBQT format for subsequent molecular docking simulations.

The docking grid for qm*Pf*DHFR was constructed based on previous studies [31], with grid box center coordinates, grid box size, and grid spacing set to cover key residues involved in antifolate inhibitor binding, as detailed in Supplementary Information (Supplementary Table S1). Molecular docking was performed using AutoDock4.2, employing a genetic algorithm (GA) with the number of GA runs set to 100, whereas the other parameters were kept at their default values [82]. To validate the docking protocol, the co-crystallized ligand WR99210 was redocked into the active site of qm*Pf*DHFR using the same parameters. The docking procedure was considered accurate and reliable when the root mean square deviation (RMSD) between the redocked and co-crystallized ligands was less than 2.00 Å [84]. Subsequently, all investigated ligands were docked according to a validated protocol. The resulting molecular docking conformations were analyzed to identify the intermolecular interactions between ligand atoms and amino acid residues using a protein–ligand interaction profiler (PLIP) [85]. The 3D structures of protein–ligand complexes were visualized and rendered using the PyMOL software v2.5.2 (Schrödinger, New York, NY, USA).

### 4.9. Molecular Dynamics (MD) Simulations

MD simulations were performed to evaluate the stability of promising compounds derived from EL and EH roots in comparison with that of WR99210 (a known qm*Pf*DHFR inhibitor) during their interaction with qm*Pf*DHFR in a simulated biological environment.

The best binding conformations of the selected ligands, including the promising compounds and reference inhibitor WR99210 in complex with qm*Pf*DHFR, served as the starting structures for the 100 ns MD simulations. These simulations were conducted to evaluate the binding stability and investigate the dynamic behavior of each qm*Pf*DHFR–ligand complex. All MD simulations were performed using AMBER18 (University of California, California, CA, USA) following previously reported protocols [86,87]. Briefly, the qm*Pf*DHFR structure was prepared using the AMBER ff14SB force field for protein charges. The protonation states of ionizable residues were assigned using PROPKA3 at pH 7.15. The ligand was described using the general AMBER force field. The partial charges of all ligands were assigned using the AM1-BCC model implemented in ANTECHAMBER. Each system was solvated in a cubic periodic box of TIP3P water molecules, ensuring a minimum distance of 10 Å between the protein surface and the box edge. Chloride ions were added to neutralize the overall charge of the system. The simulation protocol was performed with three energy minimization steps using the SANDER module in AMBER18 to eliminate steric clashes between the protein and solvent molecules and relax the system under periodic boundary conditions at a constant volume. The minimization involved 2500 steps with positional restraints applied to the protein backbone (force constant of 500 kcal/mol·Å^2^), followed by 3000 steps with a reduced restraint force constant of 10 kcal/mol·Å^2^. This was followed by a full minimization of 5000 steps using the conjugate gradient method without restraints [30]. The minimization progress was monitored based on the energy and energy-gradient values reported by AMBER18. Following minimization, each system was gradually heated from 0 K to 310 K in three sequential steps before equilibration and production runs. Each heating stage lasted for 0.5 ns: from 0 to 100 K during the first step, 100 to 200 K during the second step, and 200 to 310 K during the third step. Throughout the heating process, the backbone atoms were restrained, and the temperature was controlled using a Langevin dynamics thermostat with a collision frequency of 1 ps^−1^. The heating was conducted using an NVT ensemble. Equilibration was performed in three steps, each lasting 0.5 ns, to gradually relax the protein and surrounding solvent system before the production simulations. The SHAKE algorithm was used to constrain all bonds involving hydrogen atoms. Following equilibration under NPT conditions at 310 K, 100 ns production MD simulations were performed under the identical temperature and ensemble conditions, with the pressure maintained at 1.0 bar. Trajectory analyses were performed using CPPTRAJ to evaluate the root mean square deviation (RMSD), root mean square fluctuation (RMSF), radius of gyration (RoG), and hydrogen bond occupancy. For hydrogen bond analysis, individual interactions between ligand donor/acceptor atoms and qm*Pf*DHFR residue acceptor/donor atoms were monitored throughout the trajectories on a frame-by-frame basis. Graphs were generated using Grace v5.1.25 (Natick, MA, USA). The binding free energies (BFE) were estimated using the molecular mechanics/generalized Born surface area (MM/GBSA) method, as implemented in the MMPBSA.py module of AMBER18 [88].

### 4.10. In Silico Prediction of Drug-likeness, Pharmacokinetic, and Toxicity Profiles

**Figure 8:**
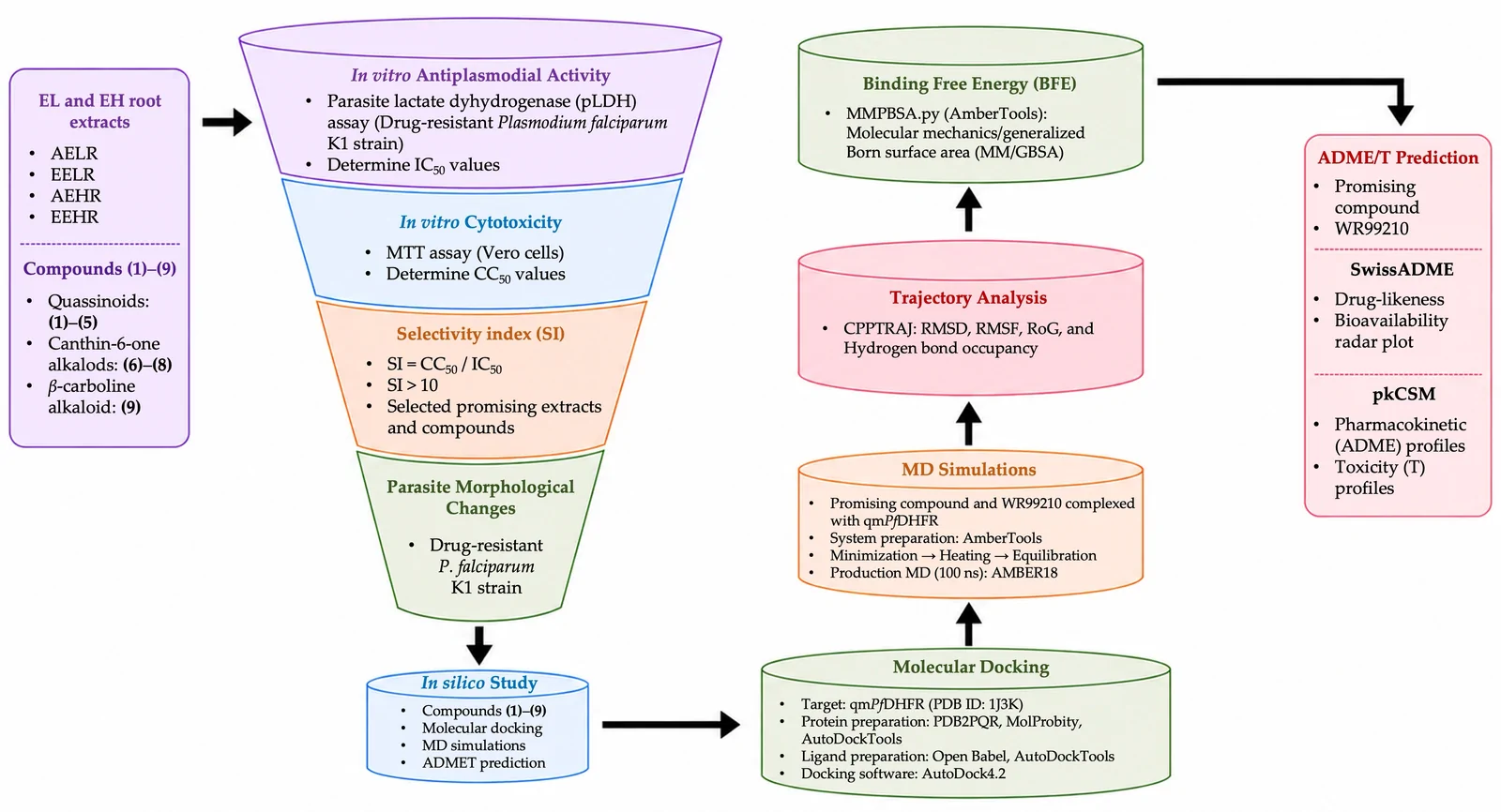
Overview of the experimental and computational workflow used in this study.

The SwissADME web tool (accessed on 14 January 2026) was used to calculate the physicochemical properties based on the Lipinski rule of five (Ro5) and to generate bioavailability radar plots of the compounds [35]. Pharmacokinetic and toxicity profiles were predicted using the pkCSM web tool (accessed on 7 August 2026) [89]. SMILES representations of compounds were obtained from the PubChem Database (https://pubchem.ncbi.nlm.nih.gov/) (accessed on 14 January 2026), and the SMILES representations were depicted in Supplementary Table S6. An overview of the experimental and computational workflow of this study is summarized in Figure 8.

### 4.11. Statistical Analysis

All experimental data were analyzed using standard descriptive statistics and are presented as mean ± standard deviation (SD). Each experiment was performed in triplicate and repeated in three independent experiments. IC_50_ and CC_50_ values were calculated using GraphPad Prism v9 (GraphPad Software, La Jolla, CA, USA).

## 5. Conclusions

This study provides the first report supporting the traditional use of EH roots for malaria treatment in Thailand. All extracts from the EL and EH roots exhibited high in vitro antiplasmodial activity against the drug-resistant *P. falciparum* K1 strain. Among these, the EEHR exhibited the greatest potency (IC_50_ = 0.51 ± 0.10 μg/mL), with low cytotoxicity toward Vero cells and a high selectivity index (SI = 62.11). Quassinoids were identified as the most bioactive constituents and exhibited excellent antiplasmodial activity (IC_50_ = 0.13–0.87 μM; SI = 6.86–206.00). In contrast, canthin-6-one and *β*-carboline alkaloids displayed good to moderate activity. All extracts, along with two promising bioactive quassinoid compounds (eurycomanone (**1**) and glaucarubolone (**5**)), disrupted *P. falciparum* K1 strain development and induced marked morphological alterations, including cytoplasmic and nuclear abnormalities. Molecular docking analysis suggested that quassinoids exhibited stronger predicted binding affinities towards qm*Pf*DHFR than alkaloids. Furthermore, the promising compound glaucarubolone (**5**) maintained favorable predicted interactions with qm*Pf*DHFR throughout the 100 ns MD simulation, although its predicted binding affinities were less favorable than that of the reference inhibitor WR99210. In addition, glaucarubolone (**5**) was predicted to satisfy key drug-likeness criteria, exhibit favorable pharmacokinetic properties, and have a low toxicity profile. Collectively, these findings highlight EH as a potential alternative source of antiplasmodial compounds comparable to the well-known EL. Based on the in silico findings, glaucarubolone (**5**) may represent a promising scaffold for further investigation towards the development of novel antimalarial agents. Further studies, including in vitro *Pf*DHFR inhibition assays, SAR analyses, and in vivo efficacy and safety evaluations, are warranted to further investigate its mechanism of action and assess its potential for development as an antimalarial lead compound.

## Figures and Tables

**Table 1:** In vitro antiplasmodial activity and cytotoxicity of plant extracts and compounds derived from EL and EH roots.

Plant Extract/Compound	pLDH Assay	MTT Assay	Selectivity Index (SI)
	*P. falciparum* K1 Strain	Vero Cells	
Aqueous extract of *E. longifolia* roots (AELR) ^a^	1.40 ± 0.46	53.81 ± 9.36	38.43
Ethanolic extract of *E. longifolia* roots (EELR) ^a^	1.21 ± 1.03	>100	>82.64
Aqueous extract of *E. harmandiana* roots (AEHR) ^a^	3.09 ± 0.77	72.41 ± 9.25	23.43
Ethanolic extract of *E. harmandiana* roots (EEHR) ^a^	0.51 ± 0.10	31.68 ± 5.75	62.11
**Quassinoid**			
Eurycomanone ^b^ (**1**)	0.14 ± 0.07	28.84 ± 6.18	206.00
13α(21)-Epoxyeurycomanone ^b^ (**2**)	0.16 ± 0.08	11.28 ± 1.73	70.50
Eurycomalactone ^b^ (**3**)	0.87 ± 0.16	5.97 ± 1.54	6.86
Chaparrinone ^b^ (**4**)	0.27 ± 0.03	6.71 ± 3.83	24.85
Glaucarubolone ^b^ (**5**)	0.13 ± 0.03	6.11 ± 2.20	47.00
**Canthin-6-one alkaloid**			
Canthin-6-one ^b^ (**6**)	47.31 ± 10.88	36.01 ± 6.11	0.76
9-Methoxycanthin-6-one ^b^ (**7**)	16.54 ± 1.16	21.95 ± 3.09	1.32
Canthin-6-one 9-O-*β*-glucopyranoside ^b^ (**8**)	20.15 ± 4.75	39.07 ± 9.76	1.93
***β*** **-Carboline alkaloid**			
*β*-Carboline-1-propionic acid ^b^ (**9**)	35.32 ± 9.20	>416	>11.77
**Antimalarial drug**			
Artesunate ^b^	0.01 ± 0.00	ND	ND
Chloroquine ^b^	0.89 ± 0.26	ND	ND
**Chemotherapeutic drug**			
Doxorubicin ^b^	ND	4.28 ± 2.81	ND

^a^ IC_50_ and CC_50_ values are expressed as mean ± standard deviation (SD) in μg/mL. ^b^ IC_50_ and CC_50_ values are expressed as mean ± SD in μM. ND: not determined.

**Table 2:** Binding energy and interacting residues of compounds derived from EL and EH root interactions with qm*Pf*DHFR, as predicted by molecular docking simulations.

Compound	Binding Energy (kcal/mol)	Hydrogen Bonds	Hydrophobic Interactions	Salt-Bridge Interactions	Inhibition Constant (K_i_)
		Residues	Residues	Residues	
**Co-crystallized ligand (qm** ***Pf*** **DHFR inhibitor)**					
WR99210	−7.64	ILE14, CYS15	LEU46, PHE58, ILE112 ^a^, LEU164	ASP54	2.53 μM
**Quassinoid**					
Glaucarubolone (**5**)	−9.07	ASP54, ASN108 ^a^, SER111	PHE58 ^b^, ILE112 ^a^	-	223.14 nM
Chaparrinone (**4**)	−8.77	ASN108 ^a^, SER111	PHE58 ^b^, ILE112	-	370.78 nM
Eurycomanone (**1**)	−8.27	ASP54, ASN108 ^a^, SER111	PHE58 ^a^, ILE112 ^a^	-	864.67 nM
Eurycomalactone (**3**)	−8.19	LEU46, ASN108 ^a^, SER111	PHE58 ^a^, ILE112	-	994.23 nM
13α(21)-Epoxyeurycomanone (**2**)	−8.04	ASP54, ASN108 ^a^, SER111	PHE58 ^a^, ILE112 ^a^	-	1.27 μM
**Canthin-6-one alkaloid**					
Canthin-6-one 9-O-*β-*glucopyranoside (**8**)	−8.28	ILE14 ^a^, ASP54, ASN108, LEU164, TYR170	VAL45, PRO113, PHE116	-	848.38 nM
9-Methoxycanthin-6-one (**7**)	−6.70	LEU46	LEU46 ^a^, ILE112 ^a^, PHE116, LEU119	-	12.34 μM
Canthin-6-one (**6**)	−6.43	LEU46	LEU46 ^a^, ILE112 ^a^, PHE116	-	19.42 μM
***β*** **-Carboline alkaloid**					
*β*-Carboline-1-propionic acid (**9**)	−7.19	ALA16, ASN108 ^a^, LEU164	LEU40 ^a^, LEU46, PHE58, ASN108	-	5.33 μM
**Antimalarial drug**					
Artesunate	−8.50	ASN108, SER167 ^a^	PHE58 ^a^, ILE112, TYR170, VAL195	-	592.47 nM
Chloroquine	−7.49	ALA16, LEU164	ALA16, LEU40, LEU46, TRP48, PHE58, ILE112, LEU164	ASP54	3.26 μM

WR99210: 6,6-dimethyl-1-(3-(2,4,5-trichlorophenoxy)propoxy)-1,6-dihydro-1,3,5-triazine-2,4-diamine. ^a^ Two interacting residues. ^b^ Three interacting residues.

**Table 3:** Molecular dynamics analysis of qm*Pf*DHFR and its complexes based on mean RMSD, RMSF, and RoG.

System	Ligand RMSD (Å)	Complex RMSD (Å)	Complex RMSF (Å)	Complex RoG (Å)
Apo qm*Pf*DHFR	—	2.14 ± 0.37	1.09 ± 0.77	18.83 ± 0.15
WR99210–qm*Pf*DHFR	0.44 ± 0.16	1.64 ± 0.34	1.06 ± 0.81	18.80 ± 0.10
Glaucarubolone (**5**)–qm*Pf*DHFR	0.63 ± 0.22	1.70 ± 0.28	1.02 ± 0.75	18.68 ± 0.14

Values are expressed as mean ± SD. —, not applicable.

**Table 4:** Binding free energy (BFE) analysis of WR99210 (qm*Pf*DHFR inhibitor) and the promising compound glaucarubolone (**5**) in the complex with qm*Pf*DHFR, as estimated using the MM/GBSA approach.

System	BFE (kcal/mol)				
	EEL	VDW	ΔG_polar_	ΔG_non-polar_	ΔG_bind_
WR99210–qm*Pf*DHFR	−18.55 ± 4.21	−44.76 ± 2.90	26.71 ± 3.11	−5.15 ± 0.25	−41.75 ± 3.41
Glaucarubolone (**5**)–qm*Pf*DHFR	−8.17 ± 8.55	−41.52 ± 4.99	17.72 ± 8.08	−4.56 ± 0.39	−36.53 ± 5.63

EEL, electrostatic interactions; VDW, van der Waals interactions; ΔG_polar_, polar solvation energy; ΔG_non-polar_, nonpolar solvation energy; ΔG_bind_, binding free energy.

**Table 5:** Predicted drug-likeness properties of WR99210 (qm*Pf*DHFR inhibitor) and the promising compound glaucarubolone (**5**).

Compound	MW (≤500 g/mol)	HBD (≤5)	HBA (≤10)	CLogP (≤5)	Rotatable Bond	Ro5 Violations
WR99210	394.68	2	4	2.68	6	No violation
Glaucarubolone (**5**)	394.42	4	8	0.07	0	No violation

MW, molecular weight; HBD, number of hydrogen bond donors; HBA, number of hydrogen bond acceptors; CLogP, calculated logarithm of the partition coefficient.

**Table 6:** Predicted pharmacokinetic properties and toxicity profiles of WR99210 (qm*Pf*DHFR inhibitor) and the promising compound glaucarubolone (**5**).

Property	Model Name	WR99210	Glaucarubolone (5)	Unit/Prediction
Absorption	Intestinal absorption (human)	80.022	63.504	%Absorbed
Absorption	P-glycoprotein substrate	Yes	Yes	(Yes/No)
Absorption	P-glycoprotein I inhibitor	No	No	(Yes/No)
Absorption	P-glycoprotein II inhibitor	No	No	(Yes/No)
Distribution	BBB permeability	−1.286	−0.585	Log BB
Metabolism	CYP2D6 substrate	No	No	(Yes/No)
Metabolism	CYP3A4 substrate	Yes	No	(Yes/No)
Metabolism	CYP1A2 inhibitor	No	No	(Yes/No)
Metabolism	CYP2C19 inhibitor	No	No	(Yes/No)
Metabolism	CYP2C9 inhibitor	No	No	(Yes/No)
Metabolism	CYP2D6 inhibitor	No	No	(Yes/No)
Metabolism	CYP3A4 inhibitor	No	No	(Yes/No)
Excretion	Total clearance	0.387	0.612	Log mL/min/kg
Excretion	Renal OCT2 substrate	Yes	No	(Yes/No)
Toxicity	hERG I inhibitor	No	No	(Yes/No)
Toxicity	hERG II inhibitor	Yes	No	(Yes/No)
Toxicity	Oral rat acute toxicity (LD_50_)	2.715	2.679	mol/kg
Toxicity	Oral rat chronic toxicity (LOAEL)	1.383	3.375	Log mg/kg_bw/day
Toxicity	Hepatotoxicity	Yes	No	(Yes/No)

BBB, blood–brain barrier; OCT2, organic cation transporter 2; hERG, human ether-a-go-go gene.

## Data Availability

The data presented in this study are available in the article and the Supplementary Materials.

## Supplementary Materials

The following supporting information can be downloaded at: https://www.mdpi.com/article/10.3390/ijms27177892/s1.
