# Supplementary material for: Antiplasmodial Compounds from Eurycoma harmandiana Pierre and Eurycoma longifolia Jack Against Drug-Resistant Plasmodium falciparum: An Integrated In Vitro and In Silico Study
Source: Int J Mol Sci. 2026 Sep 4;27(17):7892. doi: 10.3390/ijms27177892 (PMC13566563; doi:10.3390/ijms27177892)
Supplement: Supplementary file 1 [file ijms-27-07892-s001.zip › ijms-4468203-supplementary.pdf]

Supporting Information

Antiplasmodial Compounds from *Eurycoma harmandiana* Pierre and *Eurycoma longifolia* Jack Against Drug-Resistant *Plasmodium falciparum*: An Integrated In Vitro and In Silico Study

Atthaphon Konyanee [1,2], Habibah A Wahab [3,4], Ezatul Ezleen Kamarulzaman [3,5], Ahmad Marwazi Mohd Suhaimi [3], Ahmad Ghazali Ismail [3], Prapaporn Chaniad [1,2], Walaiporn Plirat [1,2], Arisara Phuwajaroanpong [2,6], Thaweesak Juengwatanatrakul [7], Tripetch Kanchanapoom [8], Gorawit Yusakul [9,10] and Chuchard Punsawad [1,2,*]

[1] Department of Medical Sciences, School of Medicine, Walailak University, Nakhon Si Thammarat 80160, Thailand; atthaphon.ko@wu.ac.th (A.K.); prapaporn.ch@wu.ac.th (P.C.); walaiporn.pi@wu.ac.th (W.P.)

[2] Center of Excellence in Tropical Pathobiology, Walailak University, Nakhon Si Thammarat 80160, Thailand; arisara.pw@wu.ac.th

[3] School of Pharmaceutical Sciences, Universiti Sains Malaysia, Minden 11800, Pulau Pinang, Malaysia; habibahw@usm.my (H.A.W.); ezatulezleen@usm.my (E.E.K.); ahmadmarwazi98@gmail.com (A.M.M.S.); ghazali.upha17@student.usm.my (A.G.I.)

[4] Malaysian Institute of Pharmaceuticals and Nutraceuticals, National Institutes of Biotechnology Malaysia, Halaman Bukit Gambir, Gelugor 11700, Pulau Pinang, Malaysia

[5] Faculty of Pharmacy, Universitas Airlangga, Kampus C UNAIR, Jl. Mulyorejo, Mulyorejo, Surabaya 60115, East Java, Indonesia

[6] School of Allied Health Sciences, Walailak University, Nakhon Si Thammarat 80160, Thailand

[7] Faculty of Pharmaceutical Sciences, Ubon Ratchathani University, Ubon Ratchathani 34190, Thailand; thaweesak.j@ubu.ac.th

[8] Faculty of Pharmaceutical Sciences, Khon Kaen University, Khon Kaen 40002, Thailand; trikan@kku.ac.th

[9] Faculty of Pharmaceutical Sciences, Naresuan University, Phitsanulok 65000, Thailand; gorawity@nu.ac.th

[10] Research and Innovation Cluster for Natural Health Products, Naresuan University, Phitsanulok 65000, Thailand

* Correspondence: chuchard.pu@wu.ac.th; Tel.: +66-75-477-411

Table of Contents

		Page
**Figure S1**	Percentage parasitemia of *P. falciparum* K1 strain-infected erythrocytes after treatment with the IC_{90} concentrations of plant extracts/compounds, compared with the 0.1% DMSO-treated control, at 0, 6, 12, 24, 36, 48, and 60 h post-treatment.	2
**Figure S2**	Root mean square deviation (RMSD) between redocked and co-crystallized conformations of 6,6-Dimethyl-1-(3-(2,4,5-trichlorophenoxy)propoxy)-1,6-dihydro-1,3,5-triazine-2,4-diamine (WR99210) within qm*Pf*DHFR crystal structures.	3
**Figure S3**	Intermolecular interactions of compounds derived from EL and EH roots with qm*Pf*DHFR.	4
**Table S1**	Grid box parameters used for molecular docking simulations of compounds derived from EL and EH with qm*Pf*DHFR (PDB ID: 1J3K).	3
**Table S2**	Hydrogen bond occupancy of WR99210 in a complex with qm*Pf*DHFR over a 100 ns production molecular dynamics trajectory.	4
**Table S3**	Hydrogen bond occupancy of glaucarubolone **(5)** in a complex with qm*Pf*DHFR over a 100 ns production molecular dynamics trajectory.	5
**Table S4**	Per-residue MM/GBSA energy decomposition of WR99210 in complex with qm*Pf*DHFR over a 100 ns MD simulation.	5
**Table S5**	Per-residue MM/GBSA energy decomposition of glaucarubolone **(5)** in complex with qm*Pf*DHFR over a 100 ns MD simulation.	5
**Table S6**	SMILES representations of WR99210 (qm*Pf*DHFR inhibitor) and the promising compound glaucarubolone **(5)**.	6
**Table S7**	Production MD simulation parameters for the WR99210–qm*Pf*DHFR and glaucarubolone **(5)**–qm*Pf*DHFR complexes.	6

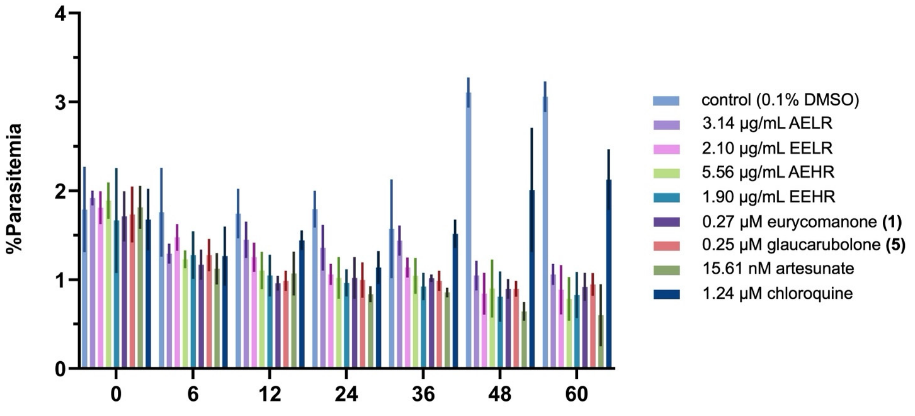

Figure S1. Percentage parasitemia of *P. falciparum* K1 strain-infected erythrocytes after treatment with the IC_{90} concentrations of plant extracts/compounds, compared with the 0.1% DMSO-treated control, at 0, 6, 12, 24, 36, 48, and 60 h post-treatment. Treatments included aqueous extract of *E. longifolia* roots (AELR) at 3.14 µg/mL; ethanolic extract of *E. longifolia* roots (EELR) at 2.10 µg/mL; aqueous extract of *E. harmandiana* roots (AEHR) at 5.56 µg/mL; ethanolic extract of *E. harmandiana* roots (EEHR) at 1.90 µg/mL; eurycomanone **(1)** at 0.27 µM; glaucarubolone **(5)** at 0.25 µM; artesunate at 15.61 nM; and chloroquine diphosphate salt at 1.24 µM. Percentage parasitemia was independently determined by two scientists under blinded conditions. Data are presented as mean ± S.D.

Table S1. Grid box parameters used for molecular docking simulations of compounds derived from EL and EH with qm*Pf*DHFR (PDB ID: 1J3K).

Protein–ligand crystal structure	Grid box size (x, y, z)	Grid box center coordinates			Grid spacing	Reference
		x	y	z		
*Pf*DHFR–WR99210 (PDB ID: 1J3K)	50 × 50 × 50	29.310	5.800	59.970	0.375	[1]

WR99210: [6,6-Dimethyl-1-(3-(2,4,5-trichlorophenoxy)propoxy)-1,6-dihydro-1,3,5-triazine-2,4-diamine].

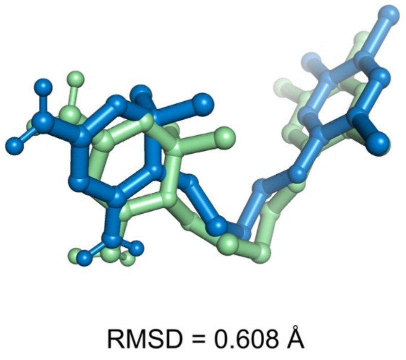

(**a**)

Figure S2. Root mean square deviation (RMSD) between redocked and co-crystallized conformations of 6,6-Dimethyl-1-(3-(2,4,5-trichlorophenoxy)propoxy)-1,6-dihydro-1,3,5-triazine-2,4-diamine (WR99210) within qm*Pf*DHFR crystal structures. The pale green conformations represent the redocked ligands, while the sky-blue conformations represent the original co-crystallized ligands.

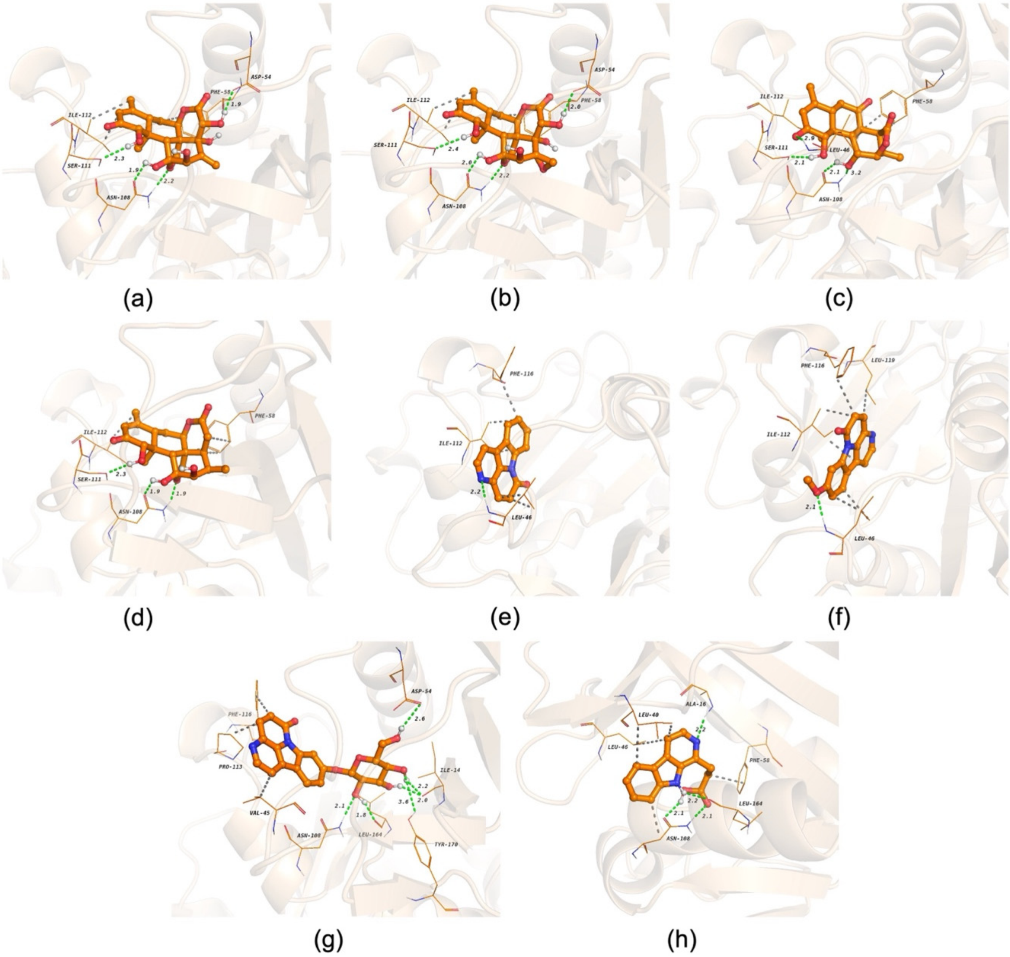

(**a**)

Figure S3. Intermolecular interactions of compounds derived from EL and EH roots with qm*Pf*DHFR. The ligands include eurycomanone **(1)** (a), 13α(21)-epoxyeurycomanone **(2)** (b), eurycomalactone **(3)** (c), chaparrinone **(4)** (d), canthin-6-one **(6)** (e), 9-methoxycanthin-6-one **(7)** (f), canthin-6-one 9-O-*β*-glucopyranoside **(8)** (g), and *β*-carboline-1-propionic acid **(9)** (h). Ligands are depicted as ball and stick models, while interacting residues are shown as line models. Heteroatoms are color-coded as follows: carbon (C), orange; oxygen (O), red; nitrogen (N), blue; and hydrogen (H), white. Green dashed lines indicate hydrogen bonds, with bond distance labeled in angstrom (Å). Gray dashed lines represent hydrophobic interactions.

Table S2. Hydrogen bond occupancy of WR99210 in a complex with qm*Pf*DHFR over a 100 ns production molecular dynamics trajectory.

Acceptor	Donor	Residue	Frames	Occupancy (%)	Average distance (Å)
UNL_232@HC4	ASN_108@ND2	ASN108	7732	7.73	2.80
UNL_232@HC13	ALA_16@N	ALA16	222	2.92	2.85
UNL_232@HC11	ALA_16@N	ALA16	2894	2.89	2.85
UNL_232@HC12	ALA_16@N	ALA16	2548	2.55	2.85
UNL_232@HN1	ILE_14@CG1	ILE14	2057	2.06	2.88
UNL_232@HN2	ALA_16@CB	ALA16	2006	2.01	2.89
UNL_232@HC2	LEU_164@CB	LEU164	1331	1.33	2.95

Table S3. Hydrogen bond occupancy of glaucarubolone **(5)** in a complex with qm*Pf*DHFR over a 100 ns production molecular dynamics trajectory.

Acceptor	Donor	Residue	Frames	Occupancy (%)	Average distance (Å)
MOL_232@CA	ASN_108@ND2	ASN108	441	0.44	2.94
MOL_232@OA	ASN_108@ND2	ASN108	429	0.43	2.94
MOL_232@OA	ALA_16@N	ALA16	103	0.10	2.94
MOL_232@OA	ASN_108@ND2	ASN108	82	0.08	2.94
MOL_232@OA	LEU_46@N	LEU46	49	0.05	2.94
MOL_232@OA	ASN_108@ND2	ASN108	48	0.05	2.94

Table S4. Per-residue MM/GBSA energy decomposition of WR99210 in complex with qm*Pf*DHFR over a 100 ns MD simulation.

	MM/GBSA										
	Residue	Van der Waals		Electrostatic		Polar solvation		Non-polar solvation		Total	
		Avg	SD	Avg	SD	Avg	SD	Avg	SD	Avg	SD
Total energy decomposition	ILE14	−1.0090	0.4600	−3.1000	0.7100	0.5100	0.2600	−0.0440	0.0170	−3.6400	0.6200
	ALA16	−1.3700	0.2900	0.1800	0.2500	0.2200	0.1800	−0.1100	0.0230	−1.0800	0.3400
	TRP48	−0.5000	0.1400	−0.1800	0.1200	0.4100	0.1600	−0.0290	0.0200	−0.2900	0.1400
	ASP54	−0.6100	0.3100	−1.8800	1.5200	3.6600	1.3700	−0.0900	0.0210	1.0900	0.5200
	PHE58	−1.8800	0.4900	−0.2800	0.2700	0.3600	0.1400	−0.2300	0.0500	−2.0400	0.5100
	ILE64	−0.0028	0.0006	−0.0130	0.0065	0.0170	0.0069	0	0	0.0009	0.0025
	SER108	−0.9600	0.3200	−0.4100	0.6900	1.6800	0.8700	−0.1300	0.0340	0.1900	0.5100
	THR185	−0.2400	0.0630	0.1700	0.1200	−0.3700	0.1300	−0.0055	0.0048	−0.4400	0.1200

Avg: Average.

SD: Standard deviation.

Table S5. Per-residue MM/GBSA energy decomposition of glaucarubolone **(5)** in complex with qm*Pf*DHFR over a 100 ns MD simulation.

	MM/GBSA										
	Residue	Van der Waals		Electrostatic		Polar solvation		Non-polar solvation		Total	
		Avg	SD	Avg	SD	Avg	SD	Avg	SD	Avg	SD
Total energy decomposition	ILE14	−0.2600	0.1800	0.0100	0.2200	−0.0008	0.2200	−0.0140	0.0240	−0.2700	0.2100
	ALA16	−0.2500	0.2900	−0.0550	0.1700	0.1100	0.1600	−0.0420	0.0410	−0.2300	0.2900
	TRP48	−0.3100	0.3000	0.0510	0.2800	0.1200	0.2500	−0.0120	0.0260	−0.1600	0.2500
	ASP54	−0.2800	0.2200	−0.8900	1.7300	1.4000	1.7900	−0.0360	0.0380	0.2000	0.1400

	**PHE58**	**−1.9300**	**0.6300**	**−0.0920**	**0.4000**	**0.1700**	**0.1900**	**−0.2400**	**0.0580**	**−2.1000**	**0.8500**
	**ILE64**	**−0.0032**	**0.0008**	**0.0160**	**0.0220**	**−0.0140**	**0.0220**	**0**	**0**	**−0.0008**	**0.0018**
	**SER108**	**−1.2800**	**0.6200**	**−0.8400**	**1.3300**	**1.1600**	**1.0610**	**−0.1200**	**0.0500**	**−1.0900**	**0.8010**
	**THR185**	**−0.0370**	**0.0230**	**0.0260**	**0.0690**	**−0.0660**	**0.0720**	**−2.05E-06**	**0.0001**	**−0.0770**	**0.0340**

Avg: Average.

SD: Standard deviation.

Table S6. SMILES representations of WR99210 (qm*Pf*DHFR inhibitor) and the promising compound glaucarubolone **(5)**.

PubChem CID	Compound	Chemical formula	Molecular weight (g/mol)	SMILES
121750	WR99210	$C_{14}H_{18}Cl_3N_5O_2$	394.7	CC1(N=C(N=C(N1OCCCOC2=CC(=C(C=C2Cl)Cl)Cl)N)N)C
441797	Glaucarubolone **(5)**	$C_{20}H_{26}O_8$	394.4	C[C@@H]1[C@@H]2[C@H](C(=O)O[C@H]3[C@@]24CO[C@@]([C@@H]1O)([C@@H]4[C@@]5([C@@H](C3)C(=CC(=O)[C@H]5O)C)C)O)O

Table S7. Production MD simulation parameters for the WR99210–qm*Pf*DHFR and glaucarubolone **(5)**–qm*Pf*DHFR complexes.

Parameter	Value
MD software	AMBER18
Number of production runs	4 consecutive runs
Duration per production run	25 ns (25,000 ps)
Total production simulation time	100 ns (100,000 ps)
Ensemble	NPT
Temperature	310 K
Pressure	1.0 bar
Thermostat	Langevin dynamics
Langevin collision frequency	1 ps^{-1}
Barostat	Berendsen
Pressure relaxation time (taup)	2.0 ps
Time step	2 fs
Non-bonded cutoff	9 Å
SHAKE	Applied to bonds involving hydrogen atoms
Periodic boundary conditions	Applied
Restraints during production	None
Trajectory recording interval	Every 1 ps
Energy/output recording interval	Every 1 ps

Reference

1. Hastuti, L. P.; Hermawan, F.; Iresha, M. R.; Ernawati, T.; Firdayani, *In-silico* studies of hydroxyxanthone derivatives as potential *pf*DHFR and *pf*DHODH inhibitor by molecular docking, molecular dynamics simulation, MM-PBSA calculation and pharmacokinetics prediction. *Inform Med Unlocked* 2024, 47, 101485.
